# Investigating Fire–Atmosphere Interaction in a Forest Canopy Using Wavelets

**DOI:** 10.1007/s10546-024-00862-0

**Published:** 2024-04-18

**Authors:** Ajinkya Desai, Clément Guilloteau, Warren E. Heilman, Joseph J. Charney, Nicholas S. Skowronski, Kenneth L. Clark, Michael R. Gallagher, Efi Foufoula-Georgiou, Tirtha Banerjee

**Affiliations:** 1 Department of Civil and Environmental Engineering, University of California, Irvine, CA 92697 USA; 2 Northern Research Station, USDA Forest Service, Lansing, MI 48910 USA; 3 Northern Research Station, USDA Forest Service, Morgantown, WV 26505 USA; 4 Northern Research Station, USDA Forest Service, New Lisbon, NJ 08064 USA; 5 Departments of Civil and Environmental Engineering and Earth System Science, University of California, Irvine, CA 92697 USA

**Keywords:** Heading surface fire, Time–frequency plane, Ramp–cliff structures, Cross-wavelet coherence, Heat/momentum fluxes

## Abstract

Wildland fire–atmosphere interaction generates complex turbulence patterns, organized across multiple scales, which inform fire-spread behaviour, firebrand transport, and smoke dispersion. Here, we utilize wavelet-based techniques to explore the characteristic temporal scales associated with coherent patterns in the measured temperature and the turbulent fluxes during a prescribed wind-driven (heading) surface fire beneath a forest canopy. We use temperature and velocity measurements from tower-mounted sonic anemometers at multiple heights. Patterns in the wavelet-based energy density of the measured temperature plotted on a time–frequency plane indicate the presence of fire-modulated ramp–cliff structures in the low-to-mid-frequency band (0.01–0.33 Hz), with mean ramp durations approximately 20% shorter and ramp slopes that are an order of magnitude higher compared to no-fire conditions. We then investigate heat- and momentum-flux events near the canopy top through a cross-wavelet coherence analysis. Briefly before the fire-front arrives at the tower base, momentum-flux events are relatively suppressed and turbulent fluxes are chiefly thermally-driven near the canopy top, owing to the tilting of the flame in the direction of the wind. Fire-induced heat-flux events comprising warm updrafts and cool downdrafts are coherent down to periods of a second, whereas ambient heat-flux events operate mainly at higher periods (above 17 s). Later, when the strongest temperature fluctuations are recorded near the surface, fire-induced heat-flux events occur intermittently at shorter scales and cool sweeps start being seen for periods ranging from 8 to 35 s near the canopy top, suggesting a diminishing influence of the flame and increasing background atmospheric variability thereat. The improved understanding of the characteristic time scales associated with fire-induced turbulence features, as the fire-front evolves, will help develop more reliable fire behaviour and scalar transport models.

## Introduction

Wildland-fire-spread behaviour in different vegetative fuel conditions is characterized to a large extent by turbulence patterns generated by the interaction between the flame and the surrounding atmosphere (Beer 1991; Clements et al. 2019; Banerjee et al. 2020; Desai et al. 2023; Banerjee 2020; Heilman et al. 2021b). In the presence of a wildland fire in a forested environment, turbulent heat and momentum fluxes within the canopy and near the canopy top play a dominant role in modifying fire-spread rates during different stages of fire-front evolution (Heilman et al. 2021b). In addition, the dispersion of smoke emissions and the transport of firebrands are modulated significantly by these patterns (Heilman et al. 2015; Goodrick et al. 2012; Koo et al. 2010). Increased turbulent mixing or diffusion of smoke near the canopy top compared to that near the surface greatly influences smoke out-flux from the canopy (Heilman et al. 2015). The transport of firebrands is dependent on their lofting by the buoyant plume and advection by the local wind, both of which are informed by spatial and temporal variations in patterns generated from the fire–atmosphere interplay (Koo et al. 2010). Improvements in our understanding of fire-induced turbulence measurements demonstrably have bearings upon improved model predictions (Clements et al. 2019; Desai et al. 2023; Bebieva et al. 2020; Dupuy et al. 2014; Andrews 2014) that inform strategies for fire management operations. For instance, fast-running simulation tools such as QUIC-Fire (Linn et al. 2020), which are used for prescribed-fire planning, rely on observation-based algorithms to compute flow fields. Predictive tools for firebrand transport and smoke dispersion also rely considerably on experimental analyses to generate outputs that are more realistic (Goodrick et al. 2012) and consistent across different existing models for a given fire event (Wadhwani et al. 2022). Quantifying the relevant temporal scales associated with processes observed during different stages of fire-front evolution is essential to model accuracy and evaluation. This necessitates a multi-scale analysis of turbulence measurements informed by fire–atmosphere interactions.

Despite the progress made in recent years (Heilman et al. 2015, 2017, 2021a; Bebieva et al. 2020; Clark et al. 2020), gaps exist in our understanding of the processes through which fire-induced turbulence redistributes heat and momentum within and above the forest canopy (Heilman et al. 2021a). While the current literature documents changes in the fraction of specific turbulent-flux events due to the passage of a fire-front, a meticulous examination of the temporal patterns of these events is currently necessary (Heilman et al. 2021a, b). Furthermore, it is known that ramp–cliff structures in the temperature signal are signatures of certain organized turbulent-flux events that contribute significantly to heat transport in forested environments during no-fire conditions (Gao et al. 1989; Paw et al. 1992; Gao and Li 1993; Katul et al. 2013). Ramp slopes (time rate of temperature increase along a ramp) have found application in the estimation of sensible heat fluxes within a forest canopy in no-fire conditions via the surface renewal analysis (Paw et al. 1995; Katul et al. 1996, 2013; Fischer et al. 2023). However, temporal variations in ramp–cliff structures resulting from the presence of fire are relatively less explored and warrant an investigation (Heilman et al. 2015; Clements et al. 2019; Goodrick et al. 2012). Moreover, traditional methods used to infer coherent structures in the presence of a fire involve Reynolds decomposition, which entails either block averaging or computing moving averages (Clements et al. 2008; Heilman et al. 2017; Desai et al. 2023). While these are useful in their own right, they have limitations arising from the sensitivity of the inferred coherent motions to the averaging window; a larger averaging window delocalizes the coherent motions in time, while a smaller window makes it difficult to separate the mean terms from the turbulent fluctuations (Desai et al. 2023). Such averaging schemes either obscure the details of the finest-scale motions that characterize fire-induced turbulence or preclude the fire-induced variability in relatively longer time scales. Given that fire-induced turbulent motions are organized across a range of scales, these limitations present a critical obstacle to our understanding of them and necessitate the employment of alternative techniques.

Wavelet transforms have the principal advantage of providing information regarding the frequency content of a process as a function of time or space (Kumar and Foufoula-Georgiou 1997). Over the last few decades, wavelet transforms have found application in exploring diverse fundamental fluid mechanics and turbulence problems (e.g., Liandrat and Moret-Bailly 1990; Meneveau 1991; Farge 1992; Rinoshika and Rinoshika 2020). One of the earliest studies to employ wavelet analysis to explore turbulence within plant canopies was conducted by Collineau and Brunet (1993a, 1993b). They applied the Mexican Hat wavelet to high-frequency (16-Hz) sonic-anemometer and thermometer data collected near the canopy top in the Les Landes forest in south-western France. The method was found to be useful in obtaining an average duration scale of approximately 29 s for ramp–cliff structures in the temperature signal (Collineau and Brunet 1993b; Raupach et al. 1996). Moreover, the method was also utilized by Watanabe (2004) in detecting ramp–cliff structures in the temperature output of large-eddy simulations (LES) conducted within and above a plant canopy. In another study, Gao and Li (1993) applied the Mexican Hat wavelet to temperature and velocity measurements taken below, at, and above the canopy height in a deciduous forest. Patterns in the wavelet coefficients of the temperature data near the canopy top, plotted on a time–scale plane, illustrated the presence of unorganized background turbulence at time scales below 10 s and more organized plume-like structures near the 50-s time scale, which merged into low-frequency patterns at longer periods associated with large-scale atmospheric turbulent motions. Alternating updraft and downdraft regions were seen from the wavelet coefficients for the vertical velocity near the canopy top, near the 50-s time scale. Qiu et al. (1995) conducted a pseudo-wavelet analysis (wherein mathematical constraints associated with a true wavelet analysis were relaxed) using the sawtooth function to identify ramp-like structures in the temperature measured in three different types of canopies. The pseudo-wavelet coefficients were utilized to obtain duration probability distributions for the ramp-like structures. The utility of wavelets in obtaining accurate estimates of turbulent fluxes was tested against the eddy covariance method by Schaller et al. (2017). The study analyzed scalar turbulent fluxes obtained from 20-Hz wind and methane concentration data collected in the flat floodplains of a river by computing cross-wavelet transforms using both, the Mexican Hat and Morlet wavelets. The Mexican Hat wavelet was able to resolve short-duration turbulent events more precisely in time, owing to its better time-localization properties. Contrarily, the Morlet wavelet was better able to classify flux events on the basis of frequencies, owing to its better frequency-localization properties. In both cases, flux estimates obtained from the cross-wavelet transform were inferred to be more accurate, since the method is devoid of the steady-state assumption required for the eddy-covariance method.

While wavelets have found extensive application in investigating atmospheric turbulence in no-fire conditions (e.g., Collineau and Brunet 1993a; Brunet and Collineau 1994; Gao and Li 1993; Qiu et al. 1995; Turner et al. 1994; Katul and Vidakovic 1996; Chen and Hu 2003; Schaller et al. 2017; Dupont et al. 2020), only a few studies have performed a wavelet-based analysis of fire-induced turbulence in vegetated environments. Seto et al. (2013) computed the frequency spectrum for the wind velocity and temperature measured during four management-scale burn experiments, conducted over different conditions of fuel and topography, using wavelet analysis. For all four burn experiments, higher energy was registered in the velocity and temperature spectra at mid and high frequencies (above $10^{-2}$ Hz) during fire-front-passage (FFP) in comparison with the pre-FFP spectra. Heilman et al. (2015) were able to identify the spectral frequency bands in which vertical velocity and streamwise velocity displayed peak energy values before, during, and after FFP for two backing surface fires of differing intensities (by ${\mathcal {O}}(1)$) in a forested environment. For the higher-intensity fire, higher energy was seen both during and post-FFP compared to the pre-FFP spectra of the streamwise and vertical velocity components in the mid to high frequencies (above $10^{-1}$ Hz). In a recent study, Katurji et al. (2022) obtained the time-integrated frequency spectra for sonic-anemometer data comprising streamwise and vertical wind velocity measurements taken at different heights along a meteorological tower during six experimental burns. Peak periods (time scales) for both velocity components ranged from 1 to 128 s across four burn plots and were found to increase with height from the fuel bed in each case.

Most of the aforementioned studies were able to indicate the peaking frequencies associated with FFP using wavelet analysis because of its ability to provide better smoothed spectral and co-spectral estimates compared to Fourier transforms in fluid flows (Addison 2017; Seto et al. 2013; Hudgins et al. 1993). However, not many studies have utilized wavelets to explore the evolution of coherent structures associated with the measured data before, during, and after FFP. Moreover, the highly transient nature of fire-induced disturbances and the presence of intermittency rationalize the application of a wavelet-based time–frequency analysis to fire-induced turbulence measurements. In this study, we apply wavelet analysis to high-frequency sonic-anemometer data collected during a heading surface fire beneath the forest canopy to analyze coherent turbulence events in the time–frequency (or time-versus-periods) domain. The following questions encompass the major research questions that we aim to answer through this work.

(i) How does the presence of a fire impact the duration and amplitude of ramp–cliff patterns typically observed in the measured temperature signal? Moreover, are these impacts uniform across all heights within the canopy?
(ii) Does the presence of a fire enhance the degree of organization of heat- and momentum-flux-bearing eddies relative to no-fire conditions? If so, at what heights within the canopy and at which time scales?
(iii) What is the relative importance of heat-flux (thermally driven) events versus momentum-flux (mechanically driven) events at different stages of the fire-front evolution? Is the Reynolds analogy violated by the presence of a fire? If so, is it more violated near the canopy top or in the canopy subspace?

## Data Overview

Tower-based meteorological data collected during an operational prescribed burn, comprising a heading surface fire beneath the canopy in the New Jersey Pinelands National Reserve (NJPNR), conducted under the U.S. Department of Defense-Strategic Environmental Research Program (SERDP) (Heilman et al. 2021b; Gallagher et al. 2023), are used in this analysis. The burn unit is located at the Silas Little Experimental Forest within NJPNR, New Lisbon, New Jersey, where the maximum canopy height ($h_\text {c}$) was approximately 20 m. On 13 March 2019, a line fire was ignited along the south-western edge of the burn unit at 1445 LT (local time = UTC − 4 h); the fireline progressed in the direction in which the ambient winds were strongest (southwesterly, $228^{\circ }$), i.e. the positive streamwise direction ($\hat{{\textbf{x}}}$). The flame length was less than 2 m, and the fireline intensity was 179 $\mathrm {kWm^{-1}}$, with a spread-rate of 1.7 $\mathrm {m\,min^{-1}}$. A network of 20-m towers was employed to collect measurements before, during, and after fire-front-passage (FFP) across these towers. These are referred to as the South, West, East, and North Towers depending on their relative location in the burn plot (Gallagher et al. 2023), written here in chronological order of the arrival of the fire-front at each tower base. Additional measurements were collected by a long-term flux tower stationed within the burn unit (Gallagher et al. 2023). Moreover, a control tower located outside the burn unit, 185 m away from its northern edge (Heilman et al. 2021b), provides information regarding the local no-fire conditions during the burn experiment. It should be noted that before the arrival of the fire-front at the North Tower, a substantial shift in the ambient winds caused them to be realigned predominantly into the positive cross-stream direction ($\hat{{\textbf{y}}}$). Therefore, the fire-front behaviour in relation to the ambient wind at the North Tower is characterized as flanking as opposed to heading at the other towers.

Data obtained from the West and Control Towers are primarily studied in this paper. The FFP time at the West Tower was reported to be 1525–1545 LT (Heilman et al. 2021b). Tower-mounted sonic anemometers measured air temperature (*T*) and east–west wind velocity, north–south wind velocity, and vertical wind velocity (*w*) components at 3 m, 10 m, and 20 m heights above ground level (AGL) at a sampling frequency of 10 Hz. Measured horizontal velocity components were rotated to obtain streamwise velocity (*u*) and cross-stream velocity (*v*) components. We denote the velocity components (*u*,  *v*,  *w*) and temperature (*T*) measured at height *h* as $u_h,~v_h,~w_h$, and $T_h$, respectively. The streamwise and cross-stream directions themselves were fixed for the entire duration of the burn experiment.

The prescribed burn experiment was conducted in the dormant season, i.e. the leaf area in the overstory vegetation was relatively low. The average plant-area density profile was highest (0.037 $\textrm{m}^{2}$m$^{-3}$) at approximately $h=12$ m (Heilman et al. 2021b). A complete description of the burn experiment, including detailed illustrations of the burn plot and the ignition line, the location of the measurement towers, the type of understory and grass vegetation, and surface fuel loading, is given by Heilman et al. (2021a, 2021b) and Gallagher et al. (2023).

**Fig. 1:**
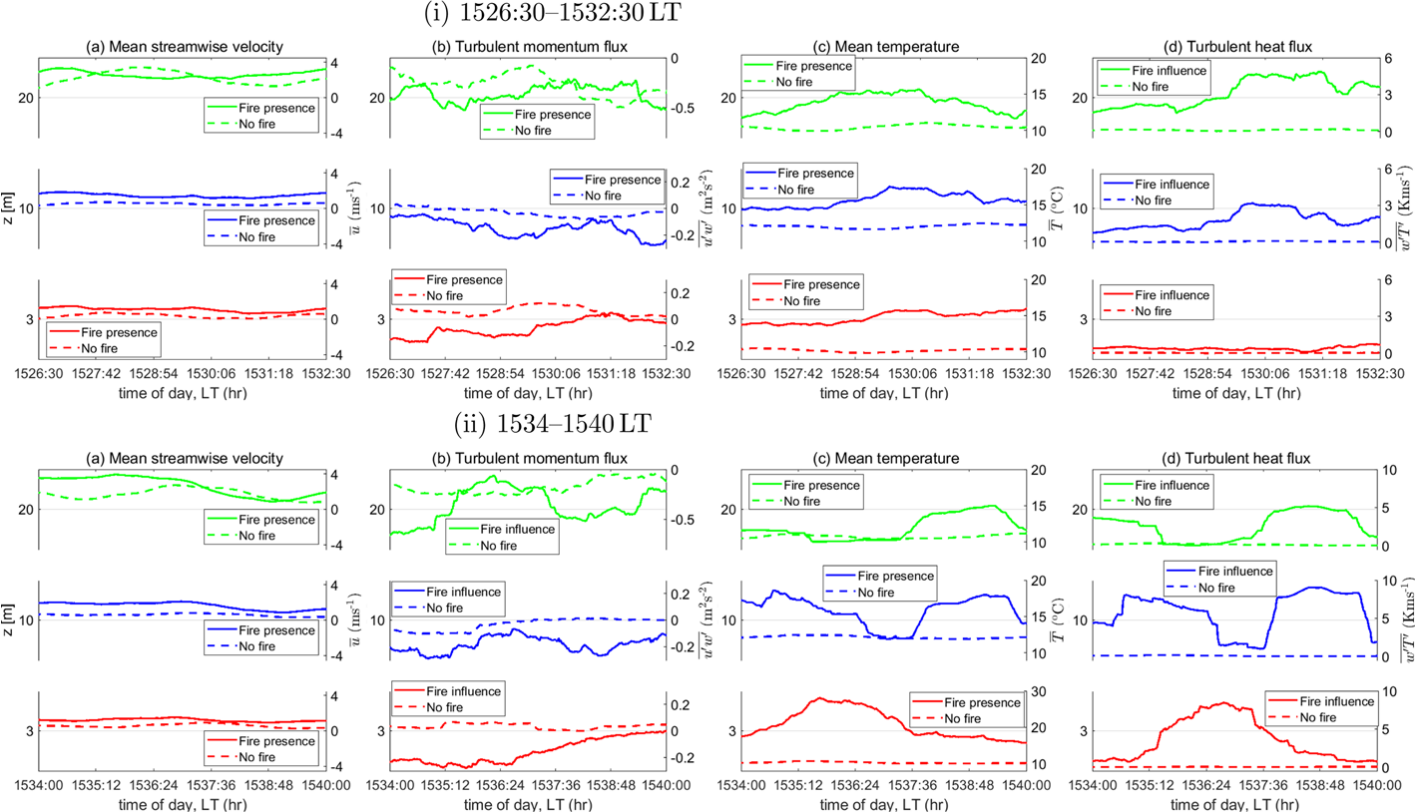
A height-wise summary of the **a** mean streamwise velocity (${\overline{u}}$), **b** turbulent momentum flux ($\overline{u'w'}$), **c** mean temperature (${\overline{T}}$), and **d** turbulent heat flux ($\overline{w'T'}$) within (i) 1526:30–1532:30 LT and (ii) 1534–1540 LT at both, the West and Control Towers. The two sub-durations fall within the stipulated FFP time

Figure 1 briefly summarizes the time evolution of the mean streamwise velocity (${\overline{u}}$), turbulent momentum fluxes ($\overline{u'w'}$), mean temperature (${\overline{T}}$), and turbulent heat fluxes ($\overline{w'T'}$) for all three measurement heights at both, the West and Control Towers, within two sub-durations that fall within the stipulated FFP time. Here, the overbar operator represents a moving average/mean (in this case, over a 2-minute moving window), while $u'$, $w'$ and $T'$ denote deviations from the mean streamwise velocity (${\overline{u}}$), the mean vertical velocity (${\overline{w}}$), and the mean temperature (${\overline{T}}$), respectively. Note that Fig. 1 is limited in its ability to provide information at scales shorter than the averaging window, as mentioned above; however, it serves to provide a brief overview of the data and as a good point of reference for our results. A detailed description of the heat-flux and momentum-flux events, including quadrant analyses, pre-, during, and post-FFP is provided by Heilman et al. (2021b), while a first-order turbulent kinetic energy budget analysis of the current data is given by Desai et al. (2023). Readers are referred to these works to obtain more context for the results presented here.

## Theory and Methodology

### Coherent Structures

Here, we first revisit the definitions of the individual heat- and momentum-flux events characteristic of the flow features in vegetative environments, which have been documented in previous works through a quadrant-analysis approach (Heilman et al. 2021a, b; Wallace 2016). Heat-flux events that are typically observed near the canopy top comprise warm updrafts ($w'>0$, $T'>0$), warm downdrafts ($w'<0$, $T'>0$), cool updrafts ($w'>0$, $T'<0$), and cool downdrafts ($w'<0$, $T'<0$). Among these, warm downdrafts and cool updrafts constitute counter-gradient motions and are typically less frequently observed. The streamwise momentum-flux events observed near the canopy top comprise sweeps ($u'>0$ and $w'<0$), ejections ($u'<0$ and $w'>0$), inward interactions ($u'<0$ and $w'<0$), and outward interactions ($u'>0$ and $w'>0$). In a forested environment, the strongest momentum-flux events near the canopy top typically comprise sweeps through which turbulence is imported into the canopy from the atmospheric surface layer (ASL) above in no-fire conditions, in relatively short time intervals (Raupach et al. 1996; Finnigan 2000). The most frequent large-scale momentum-flux events comprise ejections, which are the next most important contributors to momentum-flux events (Finnigan 2000). Generally, a coherent structure in a forested environment comprises weak ejections from the canopy top followed by a strong sweep of shorter duration into the canopy, while outward interactions and inward interactions are less frequently observed (Gao et al. 1989; Heilman et al. 2015, 2021b). It must be noted that while there is an overlap between the heat- and momentum-flux events as defined above, we treat each class of events as distinct from the other in the analysis to follow (Sect. 4.3).

Time–height cross-sections comprising isotherms of temperature deviations from the mean and fluctuations in the velocity field ($u'$, $w'$) in no-fire conditions carry important information regarding heat- and momentum-flux events within and above a canopy, their inter-relationship, and their mutual relationship with the temperature (micro)fronts depicted by the isotherms (Gao et al. 1989; Finnigan 2000). These have shown that ramp–cliff structures, both within a canopy and near the canopy top, are associated with the coherent flow features comprising predominantly of ejections and sweeps, as mentioned above (Paw et al. 1992; Gao et al. 1989; Finnigan 2000). This connection is explained with help from the description given by Gao et al. (1989) and Katul et al. (2013) in no-fire conditions, as given below. Note that the description given by Katul et al. (2013) pertains to carbon dioxide concentration in nocturnal (stable) conditions, while the explanation here pertains to temperature measurements in daytime (unstable) conditions. When a cool air parcel is swept into the canopy via a strong sweep-like downdraft from the ASL aloft in no-fire conditions, its temperature begins to increase along a ramp as it starts collecting heat from the forest surface (Katul et al. 2013). The increase in temperature along the ramp coincides with weak updrafts (mostly ejections) near the canopy top (Gao et al. 1989). The temperature of the air parcel continues to increase until the warm air parcel is finally driven out of the canopy into the ASL aloft by an ejection-like updraft. As cool air is swept into the canopy again, the measured temperature drops along a cliff. The cliff represents the sharp temperature micro-front that separates the strong sweep-like downdraft from the updraft events (Gao et al. 1989). The residence time of the air parcel within the canopy determines the time duration of the ramp structure between two consecutive occurrences of the micro-front. Ramp–cliff structures find application in the estimation of sensible heat fluxes using a method called surface renewal analysis introduced by Paw et al. (1995), which relies on the slope of the ramp structure (time rate of temperature increase along the ramp). More details regarding the procedure for accomplishing this are provided by Katul et al. (1996, 2013).

### Wavelet Transform and Analysis

#### Properties of the Continuous Wavelet Transform

The continuous wavelet transform (CWT) of a signal *g*(*t*) is defined as (Addison 2017):

1 $$\begin{aligned} W_g(a,b) = \frac{1}{\sqrt{a}}\int _{-\infty }^{\infty }g(t)\psi ^{*}\left( \frac{t-b}{a}\right) \text {d}t. \end{aligned}$$

Here $\psi (t)$ is called the “analyzing” wavelet, $\psi ^*(t)$ is the complex conjugate of $\psi (t)$, and $\psi (\frac{t-b}{a})$ is called the wavelet function. Furthermore, *b* is the location parameter, *a* is the scale parameter (also called dilation parameter), and $W_g(a,b)$ represents the wavelet coefficient associated with scale *a* and location parameter *b*. The location parameter *b* controls the temporal location of the wavelet function in the time series (Schaller et al. 2017). It must be mentioned that the characteristic frequency (*f*) associated with a wavelet of scale parameter *a* is given by $f=f_\text {c}/a$, where $f_\text {c}$ is the passband centre frequency of the analyzing wavelet’s power spectrum (Addison 2017). This implies that the characteristic frequency of a wavelet and the associated scale have a one-to-one relation, which allows us to be able to allude to them interchangeably as we have done in the remainder of this work. The relative contribution of the energy content of *g*(*t*) at scale *a* and location *b* is provided by the two-dimensional wavelet-based energy density function defined as $|W_g(a,b)|^2$ (Addison 2017). A plot of $|W_g(a,b)|^2$ in the time–frequency (or time–scale) plane is referred to as the magnitude scalogram (Addison 2017). The scalogram allows us to identify the temporal location and scale of the most energetic features of *g*(*t*), facilitating the detection of multi-scale patterns (Addison 2017; Kumar and Foufoula-Georgiou 1997). Patterns in a given frequency band can be isolated by setting the wavelet coefficients outside that band to zero and performing an inverse wavelet transform using only the wavelet coefficients in the chosen frequency band (Addison 2017). We refer to this technique as frequency- or scale-based signal reconstruction. The inverse wavelet transform is given by (Addison 2017; Kumar and Foufoula-Georgiou 1997):

2 $$\begin{aligned} g(t) = \frac{1}{C_\psi }\int _{-\infty }^{\infty }\int _{0}^{\infty }W_g(a,b)\psi _{a,b}(t)\frac{\text {d}a\text {d}b}{a^2}, \text {where}~\psi _{a.b}(t) = \frac{1}{\sqrt{a}}\psi \left( \frac{t-b}{a}\right) . \end{aligned}$$

Here $C_\psi$ is a normalizing constant.

An important characteristic of the wavelet transform comprises the differences in its time–frequency localization behaviour at different frequencies (or scales) in the time–frequency plane (Addison 2017; Kumar and Foufoula-Georgiou 1997). These differences arise from Heisenberg’s uncertainty principle, according to which, “one cannot measure with arbitrarily high resolution in both time and frequency” (to quote directly from Kumar and Foufoula-Georgiou 1997): high resolution in time will always be accompanied by low resolution in frequency and vice versa. This is typically visualized with the help of Heisenberg boxes (or cells) that constitute the time–frequency plane with time along the *x* axis and frequency along the *y* axis. High-frequency features are well-resolved in time but with higher uncertainty in their frequency localization. This manifests in the scalogram as high energy density that is diffused along the frequency axis, i.e. across multiple frequencies or scales, but is well-localized in time (taller, thinner Heisenberg boxes) when analyzing higher-frequency events. Conversely, low-frequency features are well-resolved in frequency but with high uncertainty in time localization. This manifests in the scalogram as high energy density that is diffused along the time axis, i.e. across multiple time instances, but well-localized in frequency (shorter, wider Heisenberg boxes) (Kumar and Foufoula-Georgiou 1997).

Another practical limitation of the wavelet transform comprises its boundary effects. Wavelet coefficients computed close to the boundaries of a measured (finite) signal are contaminated by the discontinuous nature of the boundaries (edges) and must be discarded in the analysis (Addison 2017). This region of “untrustworthy” wavelet coefficients becomes wider as we go higher up in scale (at lower frequencies). On the magnitude scalogram, this region manifests as a cone when the frequency axis is plotted on a logarithmic scale and is referred to as the cone of influence (Addison 2017). A detailed description of the wavelet transform along with the criteria that a function must satisfy to be classified as a wavelet, the Heisenberg boxes, the cone of influence (COI), the inverse wavelet transform, and the definition of the passband centre frequency is provided by Addison (2017) and Kumar and Foufoula-Georgiou (1997).

Finally, we add that continuous wavelet transforms are widely-used efficient tools for analyzing time series whose spectral properties vary over time. CWT analyses can be viewed as highly redundant multi-scale analyses where the scales of interest do not need to be predefined. Even if the information carried by the wavelet coefficients is strongly correlated from one scale to the next, the continuity in scale as well as time allows us to locate the features of interest along both the scale and time axes, accurately (Addison 2018). We, however, note that if the objective of the analysis is to derive a low-order parametric representation of the signal or to compress (reduce the dimensionality of) the data, redundancy is not desirable; in these cases, the use of discrete non-redundant wavelet transforms is preferable (Mallat 1999).

#### Application of the Wavelet-Based Methodology

In the following section (Sect. 4.1), patterns in the temperature signal (*T*(*t*) or simply *T*) are investigated using the magnitude scalogram (plot of $|W_T(a,b)|^2$ with time along the *x* axis and frequency along the *y* axis) obtained from the wavelet transform of *T*(*t*) at $h=3\,\text {m},~20$ m using the Mexican Hat wavelet. Given its symmetric structure, the Mexican Hat wavelet does not introduce asymmetry into the wavelet transform. The Mexican Hat wavelet prioritizes time localization over frequency localization (Addison 2017; Schaller et al. 2017), making it suitable for analyzing short-lived wide-band features such as ramp–cliff structures. Furthermore, rather than for analyzing narrow-band periodic signals, the Mexican Hat wavelet is useful for identifying abrupt discontinuities (edge detection) in the signal. An abrupt discontinuity comprising a rapid switching from a high magnitude to a low magnitude in *g*(*t*) manifests in the scalogram as a region of low (near-zero) energy density flanked on either side by regions of high energy density, which spans multiple scales (Addison 2017). Another pattern that can be identified by the Mexican Hat wavelet comprises a sudden increment followed by an exponential tail, which is called an exponential discontinuity (Addison 2017). This manifests in the magnitude scalogram as alternating regions of high and low energy density (along the time axis) that span a range of frequencies and are well-localized in time at higher frequencies but delocalized in time at lower frequencies. The *cwtft* function of MATLAB is used for the CWT, which performs the operation presented in Eq. (1) for a finite time series, and the COI is computed manually. The length of the signal used for the CWT spans 30 min (1800 s) of data: 5 min pre-FFP, 20 min during FFP, and 5 minutes post-FFP. Magnitude scalograms are represented as colour contours within the COI on the time–frequency plane. The COI is obtained by computing the wavelet coefficients for a zero function ($g(t)\equiv 0$) of the same length as *T*(*t*) and with artificially imposed step discontinuities at each edge where *g*(*t*) is set to 1. The regions of non-zero wavelet coefficients near the edges are the regions of “untrustworthy” wavelet coefficients and their inner boundaries give us the COI.

**Fig. 2:**
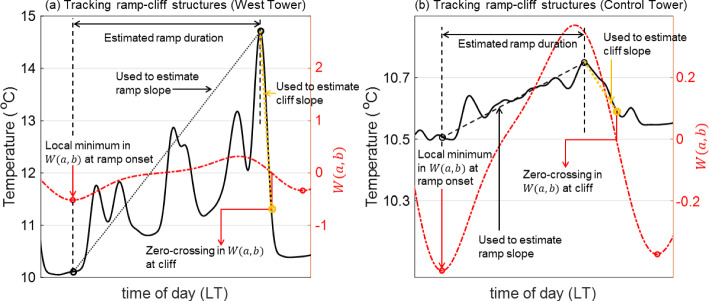
Schematic illustrations of the technique used to compute the ramp duration, ramp slope, and cliff slope of **a** a fire-modulated ramp–cliff structure at the West Tower and **b** a ramp–cliff structure in no-fire conditions at the Control Tower

In Sect. 4.2, an approximation to the original temperature signal is reconstructed in a certain frequency band using MATLAB’s *icwtft* function, which performs the operation presented in Eq. (2) for a finite time series. As mentioned above, regions of low energy density (low $|W_g(a,b)|^2$), flanked by regions of high energy density (high $|W_g(a,b)|^2$) in the scalogram represent abrupt discontinuities in *g*(*t*), when using a Mexican Hat wavelet. When the time series of the wavelet coefficients ($W_g(a,b)$) associated with an appropriate frequency/scale ($a_0$) is plotted alongside the reconstructed signal, the abrupt discontinuities correspond to the zero-crossings at which the wavelet coefficients transition from positive to negative ($W_g(a_0,b)=0$, $\frac{\text {d}W_g(a_0,b)}{\text {d}t} < 0$). This technique, referred to as “jump detection”, was utilized by Collineau and Brunet (1993b) to track cliffs (abrupt discontinuities) in the measured temperature ($g\equiv T$) in no-fire conditions. The time between subsequent “jumps” (cliffs) defined the event duration of a ramp–cliff structure. In the current study, we utilize the jump-detection technique to track cliffs in the reconstructed temperature signal. We extend this technique further to track ramps in the reconstructed temperature signal. We first note that the onset of a ramp corresponds to a trough or local minimum in the time series of the wavelet coefficients $\left( \frac{\text {d}W_T(a_0,b)}{\text {d}t} = 0\right)$. We can then trace each ramp in the reconstructed signal between a trough (local minimum) in $W_T(a_0,b)$ and the first zero-crossing that succeeds the trough where the slope is negative, i.e. the location of the immediately successive cliff. Once an individual ramp is traced, the time duration between the onset of the ramp and the peak temperature attained along the ramp in the reconstructed temperature signal can be used to quantify the ramp duration. Note that while Collineau and Brunet (1993b) computed the duration of an entire ramp–cliff structure, we compute the duration of only the ramp component. Moreover, we compute the slopes of the ramp and the cliff. The peak temperature attained along the ramp and the temperature at the onset of the ramp are used to compute the ramp slope. The peak temperature and the temperature associated with the zero-crossing mentioned above are used to compute the slope of the cliff. The application of this procedure to the reconstructed temperature signals at the West and Control Towers is illustrated in Fig. 2.

Finally, to investigate the effects of FFP on heat- and momentum-flux events, we resort to a cross-wavelet coherence analysis in Sect. 4.3. Cross-wavelet analysis between *w*(*t*) and *T*(*t*) can be used to explore the scales at which fire-induced effects on the heat flux are relevant and to assess temporal variations in the most coherent heat-flux events among those mentioned above (Sect. 3.1). Cross-wavelet analysis between *u*(*t*) and *w*(*t*) can serve a similar purpose for assessing the streamwise momentum-flux events mentioned in Sect. 3.1. We use the Morlet Wavelet to compute the cross-wavelet coherence. Unlike the Mexican Hat wavelet, the Morlet wavelet is a complex wavelet and has the advantage of giving us information from the local relative phase between the two component time series. Moreover, the Morlet wavelet provides good frequency localization, allowing for a more accurate estimation of the scales associated with turbulent-flux events (Thomas and Foken 2005; Schaller et al. 2017). Obtaining the cross-wavelet coherence is a two-step process. First, we compute the cross-wavelet transform. The cross-wavelet transform (XWT) of two time series *p*(*t*) and *q*(*t*) is defined as $W_{pq}=W_{p}W^{*}_{q}$, where $*$ denotes complex conjugation (Grinsted et al. 2004). The cross-wavelet power is defined as $|W_{pq} |$, whereas the complex argument arg($W_{pq}$) is interpreted as the local relative phase between *p*(*t*) and *q*(*t*) in the time–frequency plane. The wavelet coherence is computed in the second step. According to Torrence and Webster (1999), the wavelet coherence between two time series *p*(*t*) and *q*(*t*) as a function of scale ($R_{pq}(a)$) is defined as:

3 $$\begin{aligned} R_{pq}(a) = \frac{\text {S}(|W_{pq}a^{-1}|)}{\sqrt{\text {S}(a^{-1}|W_p|^2)\text {S}(a^{-1}|W_q|^2)}}, \end{aligned}$$

where S is a smoothing operator that comprises functions that perform smoothing along both time and scales, independently. More information about this definition is provided by Grinsted et al. (2004). For our analysis, we use MATLAB’s *wcoherence* function, which uses the analytic Morlet wavelet to return the magnitude-squared coherence ($R_{pq}^2$) as a function of time and scale. In the *wcoherence* function, a Guassian function is used for smoothing along time and a moving-average operator is used for smoothing across scales. The cross-wavelet coherence between *w* and *T* signals on one hand, and *u* and *w* signals on the other is computed near the canopy top ($h=20$ m) within two 6-min-long time windows: (i) an earlier window during FFP from 1526:30 to 1532:30 LT and (ii) later during FFP from 1534:00 to 1540:00 LT. Since the large-scale eddies associated with momentum-flux events are typically the most significant near the canopy top compared to the lower heights, we restrict our coherence analysis to $h=20$ m in this work. However, we have included the coherence analysis within the canopy subspace (at the lower heights) in the Appendix. In regions of high coherence, the local relative phase (arg($W_{wT}$) and arg($W_{uw}$)) can be used to determine the type of heat- or momentum-flux event as defined in Sect. 3.1.

## Results and Discussion

### Temperature Scalograms

**Fig. 3:**
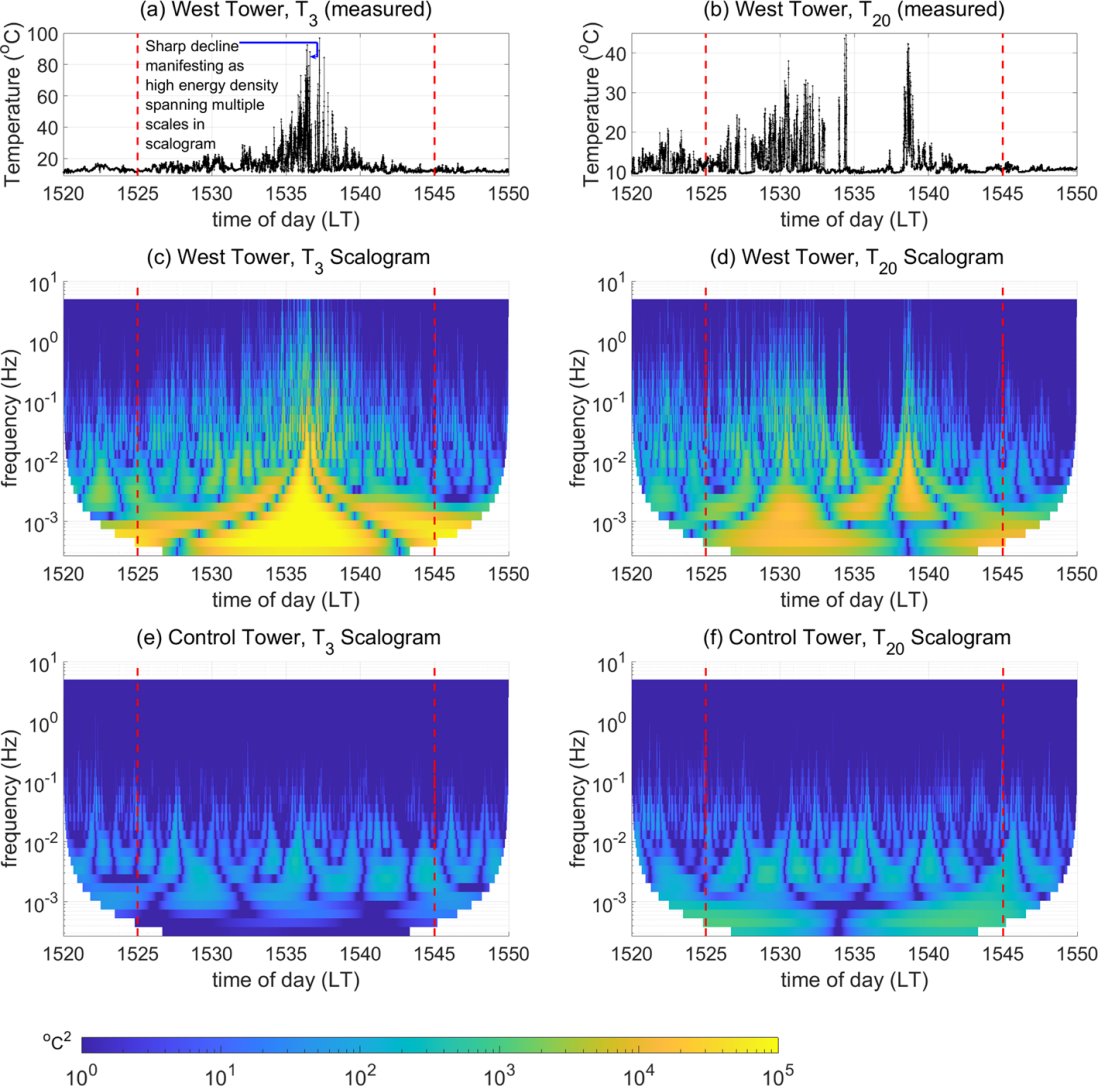
Measured temperature at the West Tower at $h=$ **a** 3 m ($T_3$) and **b** 20 m ($T_{20}$) along with the magnitude scalograms of the temperature signal at the West Tower at $h=$ **c** 3 m and **d** 20 m and magnitude scalograms of the temperature signal at the Control Tower at $h=$ **e** 3 m and **f** 20 m for the 30-min duration comprising 5 min pre-FFP, 20 min during FFP (between red dashed lines), and 5 min post-FFP at the West Tower

Figure 3 depicts the magnitude scalograms of the measured temperature signal at the West Tower and Control Towers, at $h=3$ m and 20 m, with the 20-min FFP duration delineated by the red dashed vertical lines. The measured temperature signals at the West Tower, at $h=3$ m ($T_3$) and at $h=20$ m ($T_{20}$), are shown in Fig. 3a, b, respectively. We first focus on the scalogram of $T_3$ at the West Tower. In Fig. 3c, regions of high energy density attributed to the fire influence are seen during FFP (1525–1545 LT) in the highest frequency band ($f\ge 0.1$ Hz). We compare this with the highly diminished energy density in this high-frequency band in the 5 min pre-FFP and 5 min post-FFP. This suggests that some fire-induced activity is present in the high-frequency regime ($f\ge 0.1$ Hz), where ambient atmospheric temperature perturbations are considerably weaker. It is in this high-frequency band that we expect intermittent burst-like events associated with the fire, if any, to manifest as regions of high energy density that are well-localized in time and interspersed among regions of considerably diminished energy density.

Next, a pattern of high energy density spanning a range of frequencies (multiple scales) is seen in Fig. 3c: intense yellow regions corresponding to $|W_T|^2\ge 10^4$ [$^{\circ }\text {C}^2$] that alternate with blue regions corresponding to $|W_T|^2\le 1$ [$^{\circ }\text {C}^2$] across the 20-min FFP duration at frequencies below 0.1 Hz, with the yellow regions becoming delocalized in time at lower frequencies. This pattern suggests that $T_3$ increases relatively gradually (resembling an exponential rise) to an extremely high temperature as the fire-front approaches the tower base, which is followed by a rather sharp and substantial decline. In other words, the pattern resembles a “reversed” exponential discontinuity (see Sect. 3.2.2). This pattern is also visible in the measured temperature signal ($T_3$) shown in Fig. 3a. The highest temperature value attained before the sudden decline in $T_3$ can be attributed to the presence of the fire-front close enough to the base of the tower to exert the strongest influence at $h=3$ m. Note that this will not be exactly at the location of the tower base but a short distance ($\sim$1 ft) away (Rothermel 1972), due to the tilting of the flame in the direction of the wind.

In the mid-frequency band ($10^{-2}$ Hz $<f\le 10^{-1}$ Hz), we see regions of near-zero energy density (dark blue regions) surrounded on either side by regions of high energy density (yellow regions). These regions (of near-zero energy density) represent sudden “cliff”-like switching in $T_3$ (see Sect. 3.2.2). While these cliff-like patterns are present in the measured temperature, i.e. $T_3$, across the entire 20-min FFP duration (Fig. 3a), they are more evidently seen within the duration in which the strongest temperature fluctuations are recorded near the tower base (approximately 1534–1540 LT).

We now focus on the magnitude scalogram of $T_{20}$ at the West Tower, which is shown in Fig. 3d. It is easily discerned that the overall energy density in $T_{20}$ is lower than that observed in $T_{3}$, especially across the low-frequency band ($f\le 10^{-2}$ Hz). This is expected due to the closer proximity of the flame to $h=3$ m as opposed to $h=20$ m. Moreover, the tilting of the flame in the direction of the wind results in fire-induced disturbances at the higher heights before the arrival of the fire-front at the base of the tower. This manifests as the regions of increased energy density seen in the high-frequency band ($f\ge 0.1$ Hz) earlier on (1520–1535 LT), corresponding to the increased variability in the measured temperature signal ($T_{20}$) within that duration as seen in Fig. 3b. Furthermore, large regions of highly diminished energy density (dark blue regions), interspersed by regions of high energy density (yellow regions) that are well-localized in time, are seen in the latter stages during FFP (1533–1545 LT) in the high-frequency band ($f\ge 0.1$ Hz) of the scalogram. This pattern is attributed to intermittent burst-like structures that are noticeably seen in $T_{20}$ within this time duration (Fig. 3b). Again, in the mid-frequency band ($10^{-2}$ Hz $<f\le 10^{-1}$ Hz), we see regions of near-zero energy density (dark blue regions) flanked on either side by regions of high energy (yellow regions), which are manifestations of rapid “cliff”-like switching in $T_{20}$. We expect these “cliff”-like switching patterns to be associated with ramp–cliff structures that are potentially modulated by FFP.

We now compare the magnitude scalograms for the West Tower temperature signals with those for the temperature measured at the Control Tower in Fig. 3e, f. It is immediately seen that the energy density ($|W_T|^2$) is lower than that seen for the West Tower at both heights across all the frequencies plotted in the scalograms. Moreover, in no-fire conditions, the energy density in the high-frequency band ($f\ge 0.1$ Hz), which is associated entirely with the ambient atmospheric turbulence, is considerably lower in comparison to that in the presence of a fire. However, signatures of ramp–cliff structures, associated with atmospheric (canopy) turbulence, continue to be observed in the mid-frequency band where cliffs are represented by regions of low $|W_T|^2$ flanked on either side by regions of higher $|W_T|^2$. A visual inspection shows that the cliffs are temporally further apart at the Control Tower as compared to the West Tower.

### Scale-Based Signal Reconstruction: Ramp–Cliff Structures

As discussed in Sect. 4.1, signatures of ramp–cliff patterns are observed in the mid-frequency band of the magnitude scalograms for both $T_3$ and $T_{20}$. We, therefore, reconstruct the temperature signal within time scales (periods) ranging from 3 to 90 s from the corresponding wavelet coefficients in the manner explained in Sect. 3.2.

**Fig. 4:**
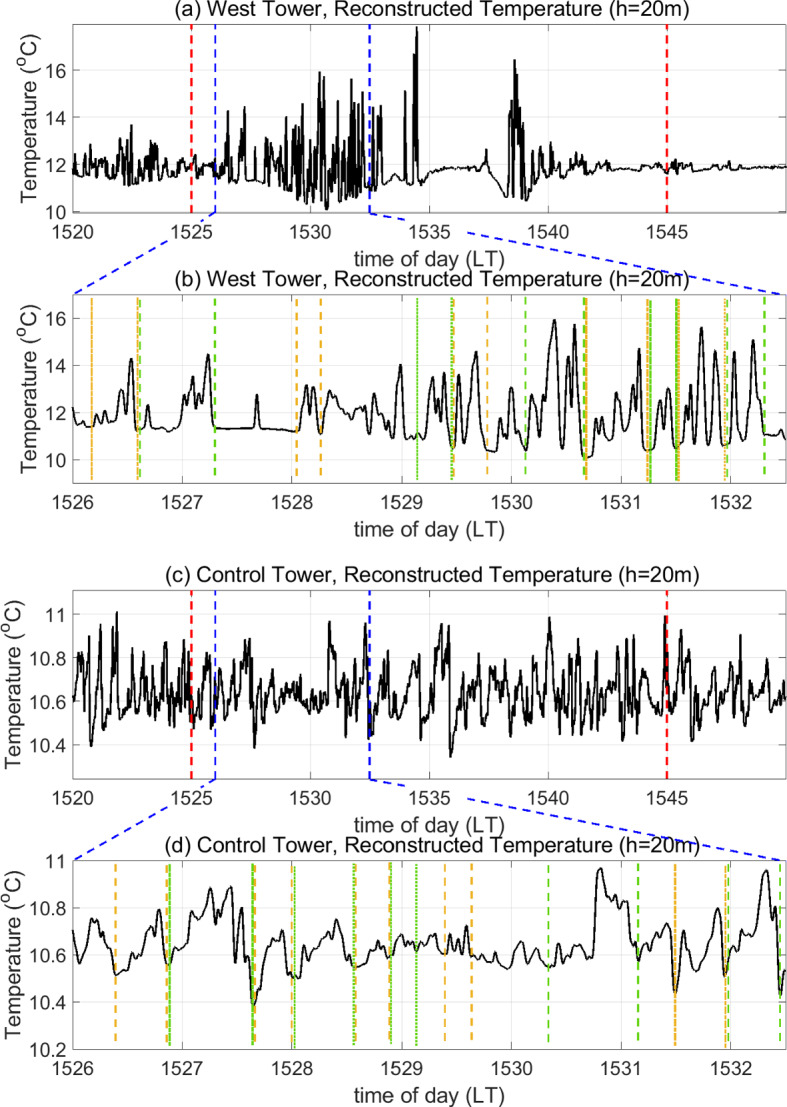
Reconstructed temperature signal at $h=20$ m in scales ranging from 3 to 90 s for **a** 1520–1550 LT and **b** 1526–1532:30 LT at the West Tower (where fire influence is experienced) as well as for **c** 1520–1550 LT and **d** 1526–1532:30 LT at the Control Tower (no-fire conditions). In panels **a** and **c**, red dashed vertical lines delineate the stipulated FFP time at the West Tower, and blue dashed lines delineate the sub-duration plotted in panels **b** and **d** respectively. In panels **b** and **d**, dashed and dash-dotted vertical lines enclose examples of ramp–cliff structures: two consecutive vertical lines with the same colour and style enclose one such observed structure. Not all structures are marked

This range includes most of the mid-frequency band and some scales from the high-frequency band, which are required to be able to detect the high-frequency component of a ramp–cliff structure, i.e. the “cliff”. The reconstruction in this range omits the time scales or periods shorter than 3 s (high-frequency fluctuations) and longer than 90 s (low-frequency turbulent motions), thereby acting as a band-pass filter. Figure 4a, b show the reconstructed temperature signal at $h=20$ m within these time scales (periods) for the entire 30-min duration at the West Tower and a shorter sub-duration of the FFP time (1526:00–1532:30 LT as shown by the blue dashed lines), respectively. Intermittent burst-like events are seen from 1534 LT onward. It appears that the effect of fire-induced turbulence is diminished between the burst-like events where the background canopy turbulence starts to become more effective. Owing to the tilting of the flame in the direction of the wind, we expect to see potential modulations in the ramp–cliff structures earlier on during FFP. We, therefore, focus on ramp–cliff patterns seen in the reconstructed temperature signal in an earlier sub-duration of the FFP time, i.e. from 1526:00 to 1532:30 LT, as shown in Fig. 4b.

**Fig. 5:**
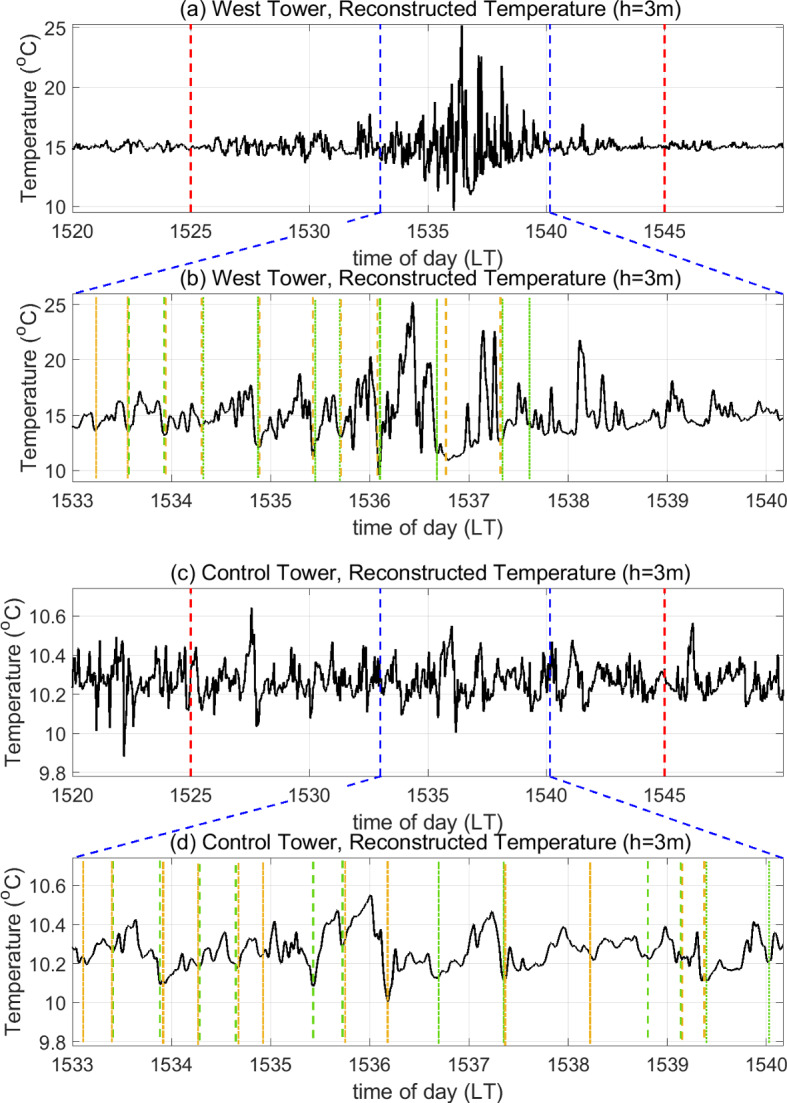
Reconstructed temperature signal at $h=3$ m in scales ranging from 3 to 90 s for **a** 1520–1550 LT and **b** 1533:00–1540:10 LT at the West Tower (where fire influence is experienced) as well as for **c** 1520–1550 LT and **d** 1533:00–1540:10 LT at the Control Tower (no-fire conditions). In panels **a** and **c**, red dashed vertical lines delineate the stipulated FFP time at the West Tower, and blue dashed lines delineate the sub-duration plotted in panels **b** and **d**, respectively. In panels **b** and **d**, dashed and dash-dotted vertical lines enclose examples of ramp–cliff structures: two consecutive vertical lines with the same colour and style enclose one such observed structure. Not all structures are marked

In Fig. 4b, some examples of ramp–cliff structures influenced by the buoyant plume (referred to as fire-modulated ramp–cliff structures here on) are delineated using dash-dotted and dashed vertical lines. It is expected that the ramp patterns during this time will be shorter in duration, while the cliffs will be steeper compared to those in no-fire conditions. To explain this hypothesis, we return to the connection between ramp–cliffs and turbulent-flux events described in Sect. 3.1. When a sweep-like downdraft brings a cool air parcel into the canopy from the ASL aloft, its temperature is expected to increase along a ramp as it collects heat from the surface fire. Since the flame acts as a stronger source of heat as compared to the forest surface under no-fire conditions, the temperature of the air parcel is expected to increase more rapidly along a ramp and up to a large magnitude. Updrafts from the buoyant plume are then expected to drive the warm air parcel out of the canopy sooner than in no-fire conditions due to their increased frequency and strength, which would cause ramp durations to be shorter than in no-fire conditions. This would then be followed by a sudden decrease in temperature from the higher magnitude attained due to the fire to ambient temperature through a cool downdraft, causing the cliff to become steeper.

With this context, we attempt to compare Fig. 4a, b with the reconstructed temperature signal at the Control Tower. Figure 4c, d show the reconstructed temperature signal near the canopy top ($h=20$ m) at the Control Tower, within time scales (periods) ranging from 3 to 90 s for the entire 30-min duration and a shorter sub-duration of the FFP time from 1526:00 to 1532:30 LT, respectively. From a visual inspection, ramp–cliff structures can be seen in the temperature signal for the entirety of the 30-min time duration shown in Fig. 4c. The drop in temperature along a cliff is less than $1\,^{\circ }$C (less steep) due to the absence of the influence of fire at the Control Tower. Some examples of ramp–cliff structures are delineated using dash-dotted and dashed vertical lines in Fig. 4d. In ramp–cliff structures of relatively higher variability, the ramps appear to be of noticeably longer duration with gradual temperature increments compared to those seen at the West Tower during this time (Fig. 4b).

Fire-modulated ramp–cliff structures can also be observed closer to the flame ($h=3$ m), as shown in Fig. 5. As seen from the reconstructed temperature signal at the West Tower (Fig. 5a), the influence of the fire is strongest at $h=3$ m from 1533 to 1540:10 LT (delineated by blue dashed lines) compared to the rest of the FFP duration. Figure 5b depicts the reconstructed temperature signal at the West Tower in this shorter sub-duration. Ramp–cliff structures with longer durations, seen from 1534 to 1535:30 LT, transform into structures with shorter durations post 1535:30 LT in Fig. 5b. In contrast, several ramp–cliff structures at this height around this time are observed to be of noticeably higher durations at the Control Tower, i.e. in no-fire conditions (Fig. 5d). Moreover, temperature increments along ramps appear to be more gradual and cliffs appear to be less steep as compared to those at the West Tower.

#### Statistical Analysis of Ramp-Like Structures

Quantitative evidence to support our hypothesis is obtained by computing a statistical distribution of the duration of the fire-modulated ramps. In order to obtain such a distribution, each ramp–cliff structure in the reconstructed temperature signal is first tracked individually using the procedure described in Sect. 3.2. Note that Collineau and Brunet (1993b) used the peak frequency from the wavelet spectrum of the measured temperature as $a_0$ (see Sect. 3.2.2) to track the ramp–cliff structures in no-fire conditions. In the current analysis, the time scale associated with the peak frequency of the wavelet spectrum falls in the low-frequency band (discussed in Appendix) and exceeds the range of time scales being explored here for ramp–cliff structures. Therefore, we resort to a much shorter time scale (higher frequency compared to the peak frequency) to obtain $a_0$. This is justified since the time scales associated with the fire-modulated ramp–cliff structures at different heights are comparatively much shorter (refer to Appendix).

**Fig. 6:**
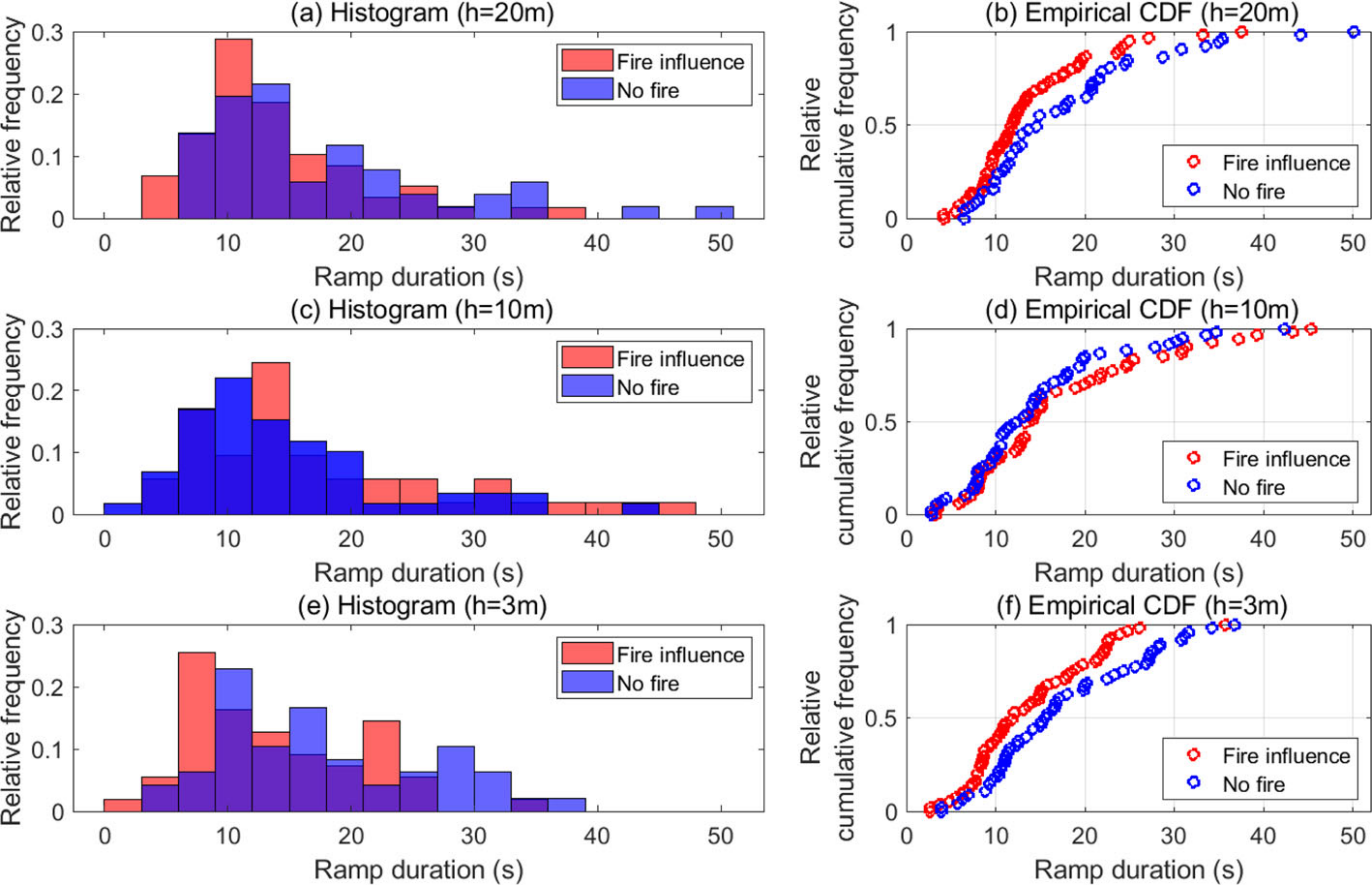
Histograms and empirical cumulative distribution functions of ramp durations associated with both, fire influence and no-fire conditions at all three measurement heights

Figure 6 shows histograms of the ramp durations obtained within each short sub-duration of the FFP time during which the fire influence is strongest near the canopy top (Fig. 6a), at the mid-canopy height (Fig. 6c), and near the surface (Fig. 6e). The corresponding empirical cumulative distribution functions (eCDFs) are plotted in Fig. 6b, d, f, respectively. The time series of wavelet coefficients ($W _T(a_0,b)$) corresponding to a time scale of approximately 23.3 s is utilized to track ramp–cliff structures. In order to obtain a sizeable sample of ramps modulated by the fire influence at each height, reconstructed temperature signals from the South, West, East, and Flux Towers are used. Ramps are concomitantly sampled from the reconstructed temperature signals at the Control Tower to obtain a duration distribution for ramps in the background no-fire canopy conditions for comparison.

Both near the surface ($h=3$ m) and the canopy top ($h=20$ m), it is seen that a higher percentage of fire-modulated ramps is associated with shorter durations as compared to no-fire conditions (Control Tower). The histograms associated with the Control Tower at these heights are characterized by thicker and/or longer tails relative to the most probable durations. It must be noted that the histograms at the Control Tower are qualitatively similar to the duration distribution of ramp structures in vegetation stands obtained using a Ramp “pseudo”-wavelet by Qiu et al. (1995) and in a pine forest obtained using the Mexican Hat wavelet by Collineau and Brunet (1993b), both in no-fire conditions. Particularly near the canopy top (Fig. 6a), where the influence of the background atmospheric canopy-scale eddies is highest in no-fire conditions, the ramp durations at the Control Tower surpass 50 s (long tail). Furthermore, near the surface (Fig. 6e), a higher percentage of ramps in no-fire conditions are seen with longer durations (thicker tail). At both heights, it is seen that the most probable durations of fire-modulated ramps are less than 10 s. The most probable fire-modulated ramp durations near the surface are observed to be shorter than those near the canopy top, due to the closer proximity of the fire to $h=3$ m resulting in more frequent and stronger fire-induced updrafts (preceding cliffs). Furthermore, the modal duration for fire-modulated ramps is lower than the modal duration for no-fire conditions at these heights, as seen from Table 1. Moreover, the mean duration of a fire-modulated ramp is approximately 4 s (20%) shorter than the mean ramp duration during no-fire conditions at these heights, while the difference amounts to more than 10 s at the 95th percentile. Shorter ramp durations also allow for a higher number of fire-modulated ramps be to sampled as compared to ramps in no-fire conditions for a given time duration, as shown in Table 1.

**Table 1:** Height-wise summary statistics of fire-modulated ramps and ramps in no-fire conditions. Tabulated modes are obtained directly from the data (not from the histograms)

Height (*h*) (m)	Fire-modulated ramps					No-fire ramps				
	No. sampled	Mean duration (s)	Modal duration (s)	95th percentile duration (s)	Mean slope ($^\circ \text {C}\,\textrm{min}^{-1}$)	No. sampled	Mean duration (s)	Modal duration (s)	95th percentile duration (s)	Mean slope ($^\circ \text {C}\,\textrm{min}^{-1}$)
20	59	13.8	5.9	26.2	15.8	51	17.8	13.0	36.4	1.1
10	53	17.1	13.3	39.0	18.8	59	14.6	10.7	32.4	1.0
3	55	13.8	8.0	24.5	27.2	48	17.6	9.3	34.9	0.8

**Fig. 7:**
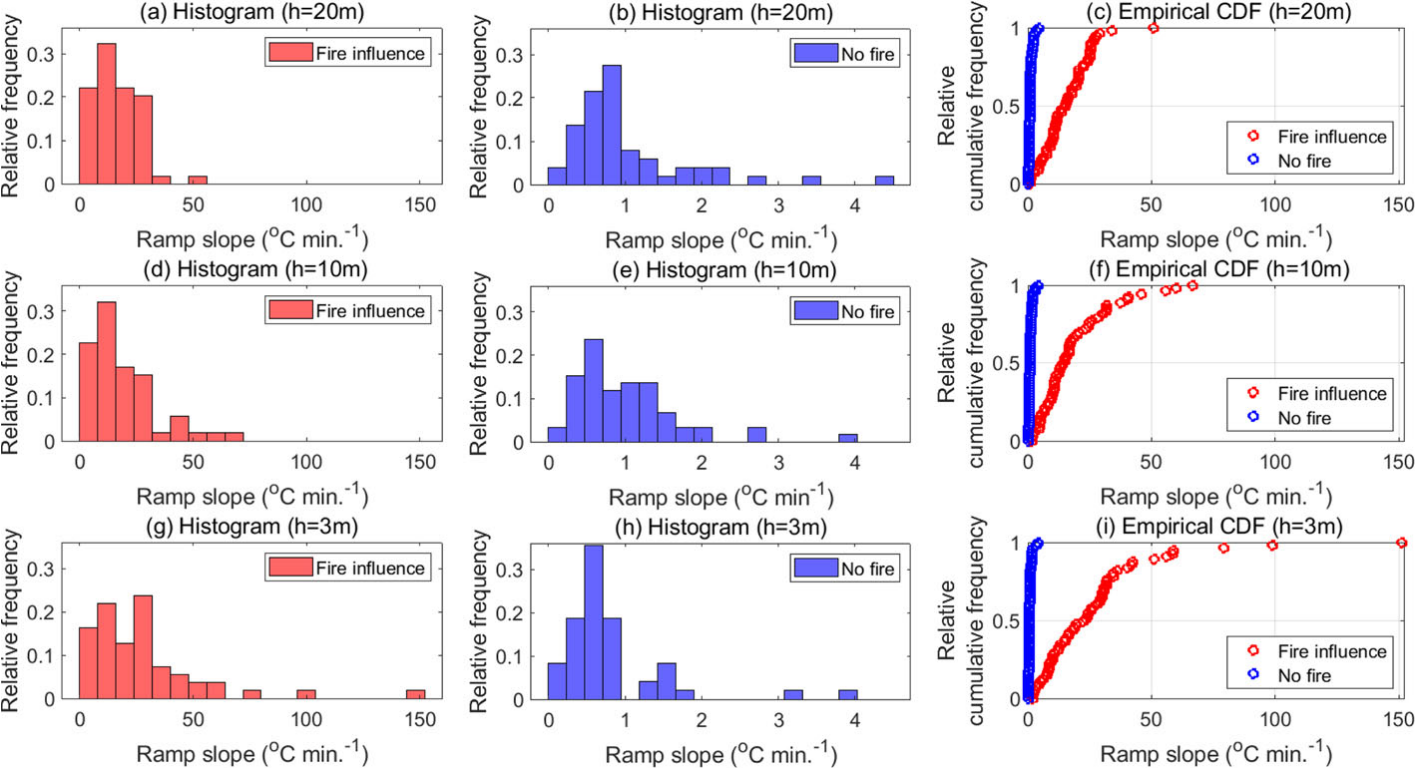
Histograms of ramp slopes associated with both, fire influence and no-fire conditions, at all three measurement heights, along with the corresponding empirical cumulative distribution functions

Interesting observations can be made for the mid-canopy height. At $h=10$ m, it is seen that the most probable durations of fire-modulated ramps (Fig. 6c) are longer than those seen near the canopy top and the surface. In fact, near the tail end of the histogram, fire-modulated ramps with durations longer than 40 s are also seen as outliers. Moreover, Fig. 6d shows that a higher percentage of fire-modulated ramps are associated with longer durations than ramps in no-fire conditions. This is symptomatic of intermittency at the mid-canopy height. Long spells comprising weak sweep-like downdrafts that are attributed to the background canopy turbulence between strong and sudden fire-induced temperature fluctuations result in longer effective fire-modulated ramp durations. This suggests that in the time duration when the fire influence is strongest mid-canopy, the background canopy turbulence is also quite effective at this height. Given that the influence of background canopy-scale eddies that originate near the canopy top is known to extend down to the mid-canopy height in no-fire conditions (Raupach et al. 1996), it is expected for the fire influence and background canopy turbulence to interact in this manner at this height. Furthermore, as mentioned in Sect. 2, leaf area was relatively low in the overstory during this burn experiment. This would have allowed for higher accessibility of the background canopy-scale eddies to the mid-canopy height, reinforcing their influence at $h=10$ m.

It must be noted that ramp durations are not the only measure of the fire influence at different heights. As seen above, it is possible to track ramps in no-fire conditions at $h=10$ m, which have relatively shorter periods than fire-modulated ramps. However, the increase in temperature along such ramps is considerably low, so that their contribution to the heat flux is relatively insubstantial. Therefore, we need to consider another measure of the influence of the fire-front, i.e. the ramp slope. Histograms and eCDFs of the slopes of fire-modulated ramps and ramps in no-fire conditions at all three heights are shown in Fig. 7. The histograms show that slopes of fire-modulated ramps near the canopy top can go up to 50 $^\circ$C $\mathrm{min.}^{-1}$ (Fig. 7(a)) and of those near the surface can go up to 150 $^\circ$C $\mathrm{min.}^{-1}$ (Fig. 7(g)), while ramp slopes in no-fire conditions are bounded by 5 $^\circ$C $\mathrm{min.}^{-1}$ (Figs. 7(b) and 7(h)). At $h=10$ m, it is seen that the most probable ramp slopes under fire influence (Fig. 7(d)) are considerably higher than in no-fire conditions (Fig. 7(e)). This captures the effect of strong and sudden upsurges in temperature induced by the fire even as the influence of the background canopy turbulence between these upsurges causes the effective ramp periods to increase. The eCDFs indicate that a higher percentage of fire-modulated ramps are associated with higher slopes as compared to ramps in no-fire conditions at all heights. Furthermore, as seen from Table 1, the ratio of the mean slope of a fire-modulated ramp to the mean slope of a ramp in no-fire conditions is highest near the surface (approx. 30) and decreases with height to about 14 near the canopy top due to decreasing proximity to the flame. This analysis suggests that fire-modulated ramps are characterized by relatively shorter durations and steeper increments to high temperatures as compared to no-fire conditions. Moreover, the values summarized in Table 1 can be utilized to compute sensible heat fluxes by invoking the surface renewal method (Appendix).

#### Statistical Analysis of Cliff-Like Structures

**Fig. 8:**
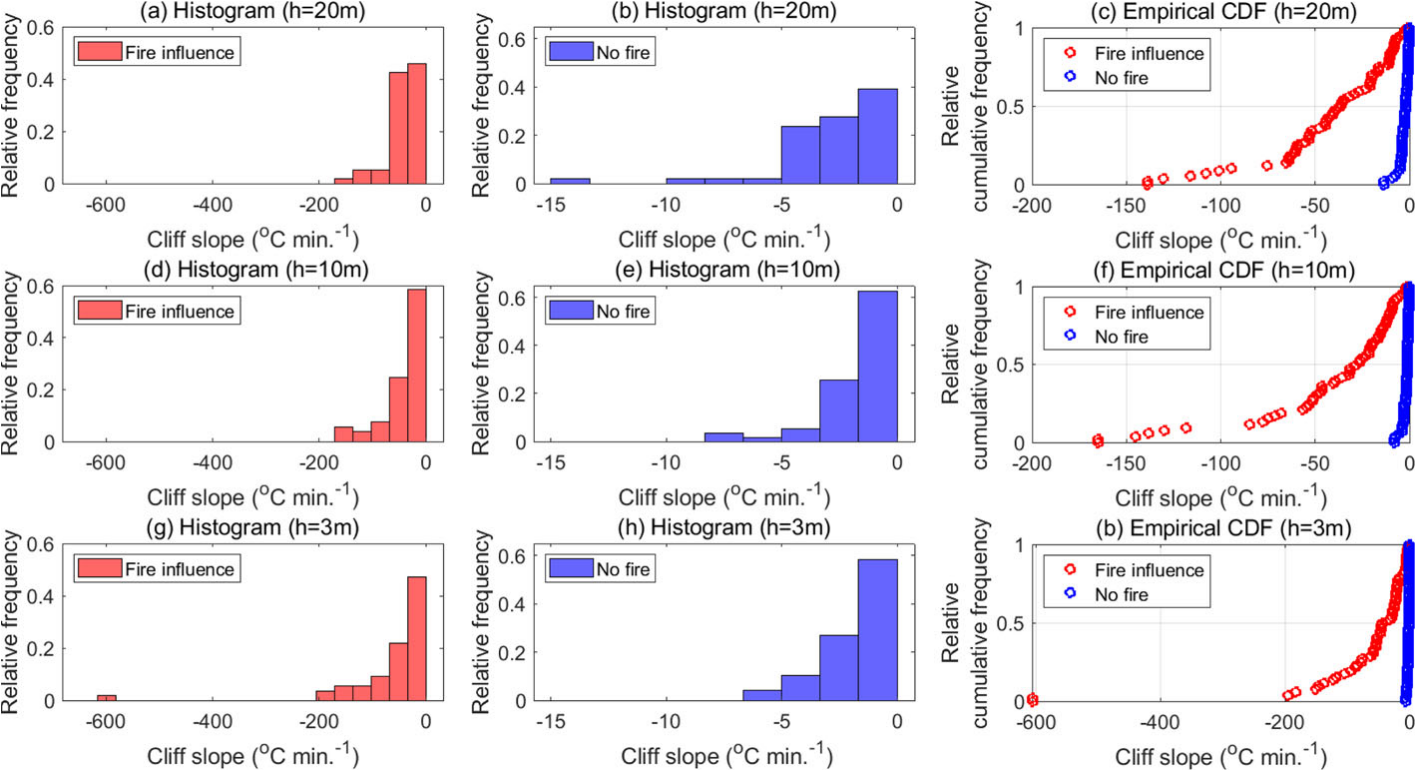
Histograms of cliff slopes associated with both, fire influence and no-fire conditions, at all three measurement heights, along with the corresponding empirical cumulative distribution functions

Figure 8 shows the histograms and eCDFs of cliff slopes under fire influence and no-fire conditions. The histograms show that fire-modulated cliff slopes can reach magnitudes that are an order of magnitude higher compared to those of cliff slopes in no-fire conditions at all three heights. The eCDFs show that near the canopy top and the flame, approximately 50% of fire-modulated cliff slopes are above 35 $^\circ$C$\,\text {min}^{-1}$ in magnitude (Fig. 8c). At $h=10$ m, approximately 50% of fire-modulated cliff slopes are above 25 $^\circ$C$\,\text {min}^{-1}$ in magnitude (Fig. 8f), which is much steeper than the entire range of no-fire cliff slopes at all three heights. In fact, near the flame ($h=3$ m), the highest observed cliff-slope magnitude is approximately 600 $^\circ$C$\,\text {min}^{-1}$ (Fig. 8g, i), which represents a decline of approximately 10 $^\circ$C in one second. We expect this steep slope to correspond to the sudden cooling that occurs after the fire-front has just passed by the tower base. Additionally, Table 2 shows that the ratio of mean fire-modulated cliff slopes to mean no-fire cliff slopes is highest near the surface (or flame) where it exceeds 30; this ratio decreases with height to approximately 15 near the canopy top. This is due to the closer proximity of the flame to $h=3$ m which causes the temperature to rise to relatively higher values before undergoing a sudden drop to ambient conditions, as compared to the higher heights.

**Table 2:** Height-wise summary statistics of the slopes of fire-modulated cliffs and cliffs in no-fire conditions

Height (*h*) (m)	Fire-modulated cliffs			No-fire cliffs		
	No. sampled	Mean slope ($^\circ \text {C}$ $\textrm{min}^{-1}$)	Median slope ($^\circ \text {C}$ $\textrm{min}^{-1}$)	No. sampled	Mean slope ($^\circ \text {C}$ $\textrm{min}^{-1}$)	Median slope ($^\circ \text {C}$ $\textrm{min}^{-1}$)
20	59	$-40.6$	$-36.4$	51	$-2.6$	$-2.0$
10	53	$-40.4$	$-26.8$	59	$-1.7$	$-1.2$
3	55	$-61.7$	$-38.1$	48	$-1.7$	$-1.2$

Since the drop in temperature along a cliff at a certain height is correlated to the influx of cool air via cool downdrafts, it follows that the slope of these cliffs must be associated with the strength of the cool-downdraft events. Near the surface, the presence of the flame not only results in an increased rate of temperature increase along a ramp but also induces strong buoyant updrafts causing an increased up-flux of warm air at $h=3$ m. This upward mass flux is compensated by a down-flux of cool air during the cool-downdraft event that follows. Since the cool-downdraft event is shorter in duration compared to the warm-updraft event, the compensatory down-flux of cool air must be stronger, compared to no-fire conditions, within that short time interval. This is also in line with the increased magnitude of turbulent fluxes associated with cool downdrafts near the surface, relative to no-fire conditions, observed by Heilman et al. (2021a) for a high-intensity backing surface fire beneath the canopy. Near the canopy top, the presence of the buoyant plume induces stronger warm updrafts, compared to no-fire conditions, which are followed by stronger cool-downdraft events; a similar explanation can be provided for the increased strength of the stronger sweep-like downdrafts (relative to no-fire conditions) that import cool air into the canopy from the ASL aloft. However, lesser proximity from the flame at this height causes the cliffs to be less steep compared to fire-modulated cliffs near the surface.

### Cross-Wavelet Coherence Analysis: Turbulent Fluxes

#### Heat- and Momentum-Flux Events

**Fig. 9:**
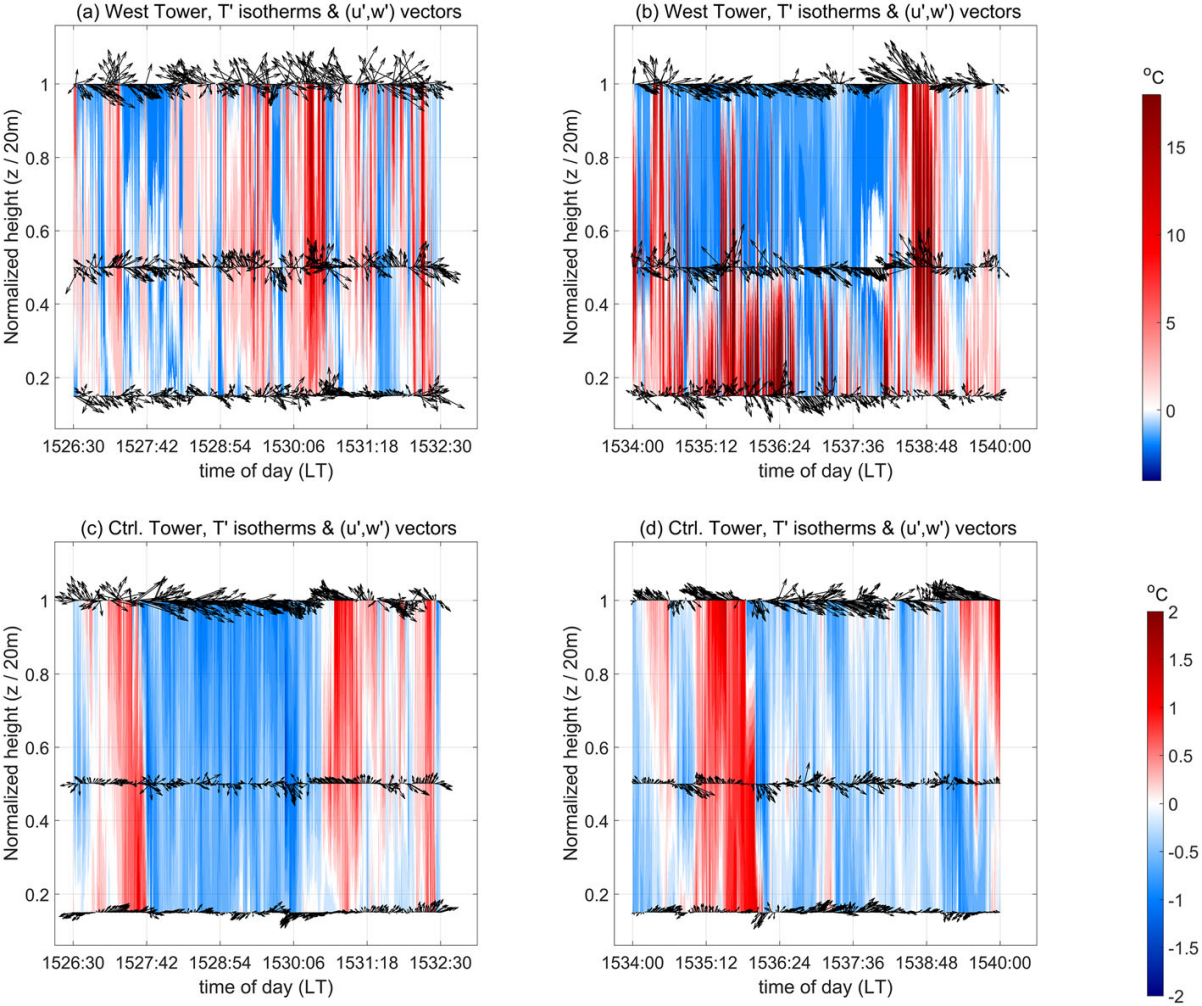
Time–height cross-sections, comprising isotherms (filled) of temperature fluctuations ($T'$) overlaid with vector arrows of $u'\hat{{\textbf{x}}}+w'\hat{{\textbf{z}}}$, at the West Tower from **a** 1526:30–1532:30 LT and **b** 1534–1540 LT, and at the Control Tower for **c** 1526:30–1532:30 LT and **d** 1534–1540 LT

In order to establish some context for the cross-wavelet coherence analysis, we first attempt to identify heat- and momentum-flux events in the current scenario as defined in Sect. 3.1. Figure 9a–d show time–height cross-sections comprising isotherms of temperature fluctuations ($T'$), which are represented by the colour contours, overlaid with arrows that represent the vector sum of the streamwise horizontal and vertical velocity fluctuations ($u'\hat{{\textbf{x}}}+w'\hat{{\textbf{z}}}$) at the West and Control Towers, respectively. Here, fluctuating quantities ($u_i',~T'$) are obtained by subtracting one-hour moving means ($\overline{u_i},~{\overline{T}}$) from the corresponding measured quantities: $u_i'=u_i-{\overline{u}}$, $T'=T-{\overline{T}}$ (as done by Desai et al. 2023). The time–height cross-sections are depicted for two 6-minute time windows: (i) 1526:30–1532:30 LT during which the influence of the fire is strongest near the canopy top due to the tilting of the flame in the direction of the wind and (ii) 1534–1540 LT, during which the flame is closest to the base of the West tower as discussed before. The three heights above the fuel bed are normalized by the maximum canopy height ($h_\text {c}=20$ m). Contours of positive temperature fluctuations (warm) are depicted in shades of red, while those of negative temperature fluctuations (cool) are depicted in shades of blue. These figures are inspired by the study by Gao et al. (1989) on organized turbulence structures within and above the canopy in no-fire conditions as mentioned in Sect. 3.1.

As seen in Fig. 9a–b, strong temperature microfronts comprising warm isotherms (shades of red) followed immediately by cool isotherms (shades of blue) pass by the different measurement heights at the West Tower. Owing to the sudden and strong temperature fluctuations ($T'$) induced by the presence of the fire, the warming temperature microfronts are considerably strong and frequently observed to last for relatively short time durations. This is more noticeable near the canopy top ($z/h_\text {c}=1$) from 1526:30 to 1532:30 LT (Fig. 9a) than from 1534 to 1540 LT (Fig. 9b), due to the enhanced influence of the fire near the canopy top in the earlier time window. This suggests that fire-induced coherent heat-flux events or eddies must also be active (or organized) at relatively shorter time scales compared to no-fire conditions. Contrarily, at the Control Tower (Fig. 9c, d), low-temperature fronts (shades of blue) are observed to persist for longer durations and high-temperature fronts (shades of red) are seen to be relatively weaker owing to comparatively weaker temperature fluctuations ($|T'|<2^{\circ }$C). Therefore, the most coherent heat-flux events or eddies in no-fire conditions are expected to be active (or organized) mainly at relatively longer time scales. This can also be observed from the more “well-behaved” pattern of warm updrafts and cool downdrafts that persist for relatively longer durations as they occur near the canopy top ($z/h_\text {c}=1$) at the Control Tower.

**Fig. 10:**
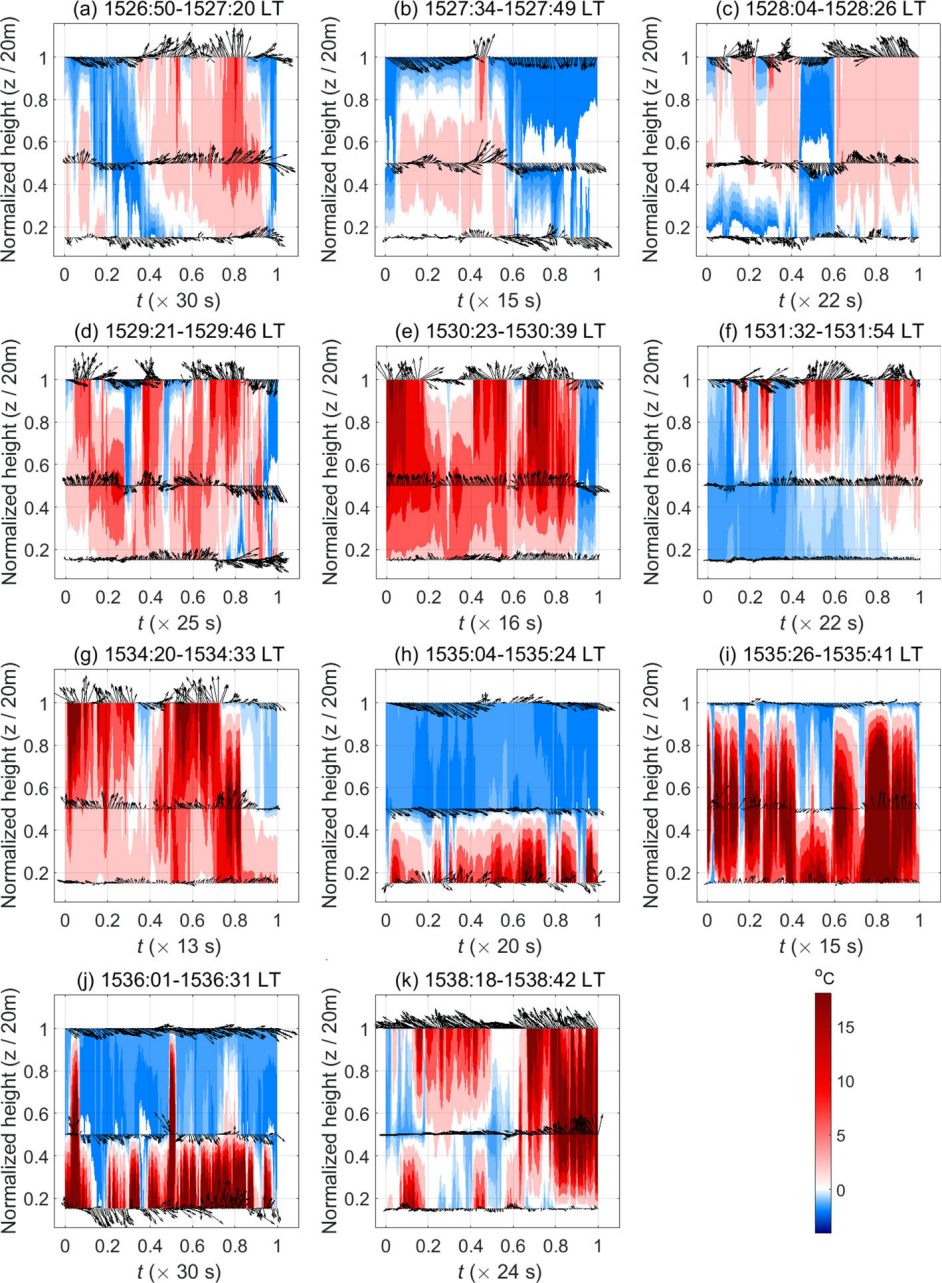
Normalized time–height cross-sections comprising isotherms (filled) of temperature fluctuations ($T'$), overlaid with vector arrows of $u'\hat{{\textbf{x}}}+w'\hat{{\textbf{z}}}$, during different short time windows within the FFP time at the West Tower. Panels **a**–**f** fall within 1526:30–1532:30 LT and panels **g**–**k** fall within 1534–1540 LT

Given the variability in the temperature and velocity signals during FFP, it is difficult to see the correlation between the temperature microfronts and the velocity vectors at the West Tower from Fig. 9a, b. We, therefore, present “slices” of normalized time–height cross-sections for the West Tower in Fig. 10. Each “slice” is of a sufficiently short duration to be able to assess possible heat- and momentum-flux events and the correlation as mentioned above. Figure 10a–f fall within the earlier time window (1526:30–1532:30 LT), whereas Fig. 10g–k fall within the latter time window (1534–1540 LT). Near the canopy top, cooler temperatures are mostly associated with downdraft events ($w'<0$) while warmer temperatures are mostly associated with updraft events ($w'>0$). When the influence of the fire begins to be experienced at the higher heights (Fig. 10a, b), persistent cool downdrafts and sweeps (of high-momentum wind) start to concede to warm updrafts and outward interactions (of high-momentum wind) and ejections (of low-momentum wind). Thereafter (Fig. 10c), cool downdrafts and sweeps switch back and forth with warm updrafts and outward interactions at shorter time scales before being followed briefly by cool downdrafts and inward interactions (of low-momentum wind), and then by more persistent warm updrafts and ejections. As the flame advances toward the tower base (Fig. 10d–f), the buoyancy-driven effects of the tilted flame intensify at the higher heights causing warm updrafts, ejections, and outward interactions to occur more frequently at the expense of cool downdrafts and sweeps; nevertheless, some cool downdrafts and inward interactions are also noticeably observed during this time (Fig. 10d, e). Both the increased strength and occurrence frequency of warm updrafts near the canopy top against typical no-fire conditions suggest that buoyancy-driven heat-flux events have considerably high variability during these times. Note that counter-gradient heat-flux motions are also briefly observed including cool updrafts in Fig. 10a, d–f, as well as warm downdrafts in Fig. 10c, e, f. However, these are short-lived and mostly occur as warm updrafts transition to cool downdrafts and vice versa.

As the fire approaches the tower base (after 1534 LT), warm temperature isotherms tend to coincide more noticeably with warm updrafts and ejections near the canopy top (Fig. 10g) and cooler temperature isotherms with cool downdrafts and sweeps. Strong and persistent sweeps and cool downdrafts, associated mainly with the background canopy turbulence, interspersed by weak sporadic outward interactions and cool updrafts (counter-gradient heat-flux motions) start to be seen near the cooler canopy top (Fig. 10h). When the fire-front is sufficiently near the base of the tower to induce the strongest temperature fluctuations at $h=3$ m (Fig. 10i), strong updrafts are seen in the warm mid-canopy region ($z/h_\text {c}=0.5$). These coincide with persistent sweeps and cool downdrafts near the canopy top that are relatively weaker due to the resistance provided by the strong updrafts from the mid-canopy height. A “hiatus” period then sets in near the canopy top where the cool downdrafts and sweeps continue to prevail (Fig. 10j). Finally, as the fire-front departs from the tower base, strong warm updrafts, likely associated with residual combustion, and ejections are seen near the canopy top (Fig. 10k). Since the stronger warming temperature fluctuations are experienced more frequently near the tower base compared to the canopy top after 1534 LT, we expect to see an overall diminishing influence of heat-flux events and an increasing influence of momentum-flux events near the canopy top during this latter stage of FFP (latter time window) compared to the earlier time window. Summarily, cool downdrafts, warm updrafts, sweeps, and ejections seem to be the more frequently observed events near the canopy top.

#### Cross-Wavelet Coherence

**Fig. 11:**
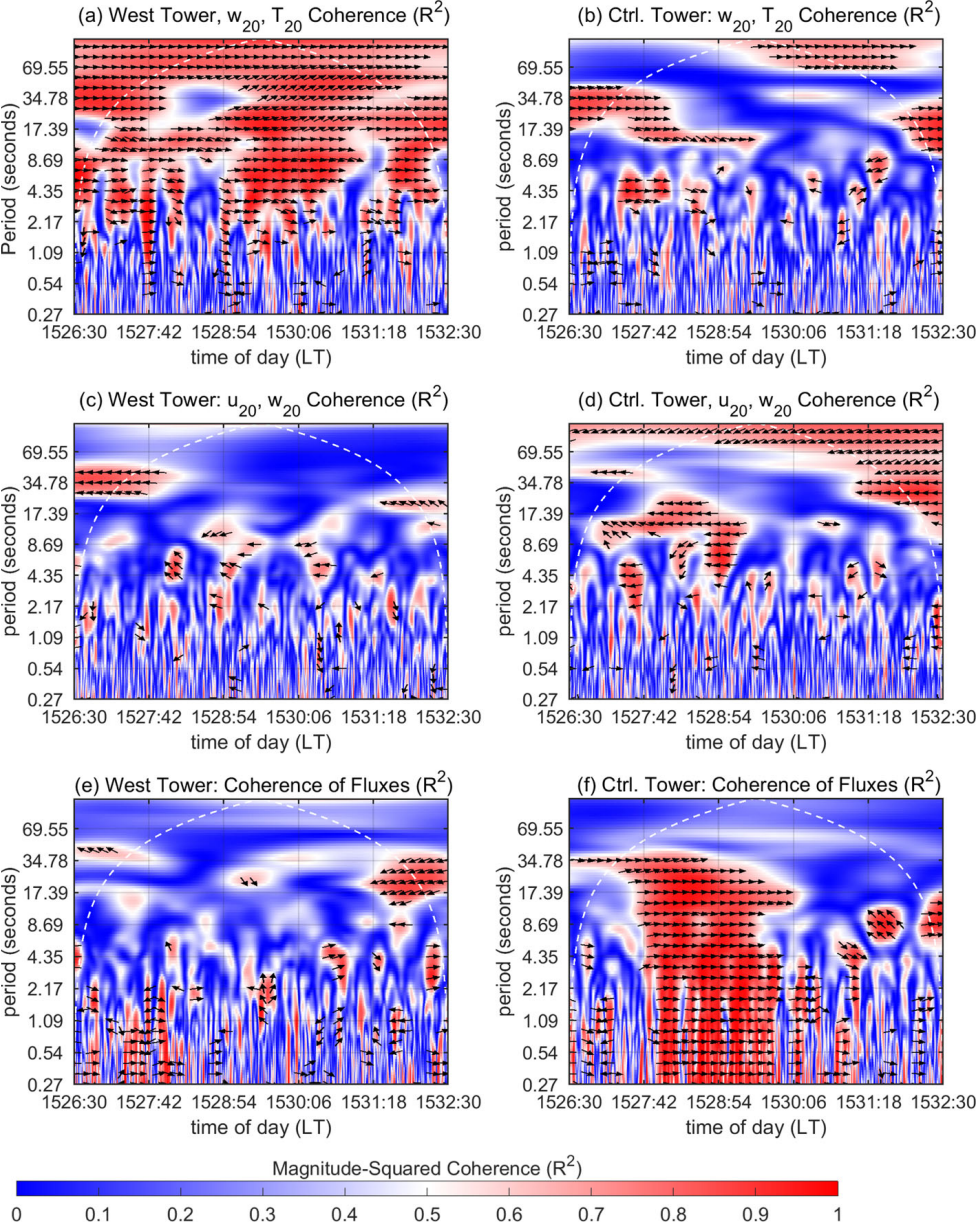
Magnitude-squared coherence ($R^2$) near the canopy top, i.e. at $h=20$ m, for the 6-min time window 1526:30–1532:30 LT. **a** $R^2_{wT}$, **c** $R^2_{uw}$, and **e** $R^2$ between $w'T'$ and $-u'w'$ at the West Tower. **b** $R^2_{wT}$, **d** $R^2_{uw}$, and **f** $R^2$ between $w'T'$ and $-u'w'$ at the Control Tower. Phase arrows are shown for $R^2\ge 0.6$ in all cases: rightward-pointing arrows indicate in-phase motions; leftward-pointing arrows indicate motions out of phase by $\pi$ radians. The white dashed line represents the cone of influence (COI) in all cases

We now turn our attention to Fig. 11, which depicts $R^2_{wT}$ and $R^2_{uw}$ along with phase arrows for $R^2\ge 0.6$ at the West and Control Towers. These are shown at $h=20$ m for the 6-min time window 1526:30–1532:30 LT. Time of day (*t*) starting from 1526:30 is plotted along the *x* axis, with the corresponding time periods (scales) along the *y* axis. At the West Tower (Fig. 11a), several regions of high $R^2_{wT}$ (shades of red) are seen spanning periods from 1 s to 1 min for this time duration. Note that during warm updrafts and cool downdrafts, turbulent fluctuations in *w* and *T* are in phase (by definition). Contrarily, these are out of phase during counter-gradient motions. The phase arrows in the region of high $R_{wT}^2$ indicate that the measured signals $w_{20}$ and $T_{20}$ are in phase (rightward-pointing arrows) for the most part. This suggests that the regions of high $R^2_{wT}$ are signatures of warm updrafts and cool downdrafts. The high $R^2_{wT}$, for $t\le$ 1527:06 LT, at relatively longer periods (above 9 s), is associated with cool downdrafts that persist for more than 9 s in Fig. 10a. The high $R^2_{wT}$ for $t\approx$ 1527:06 LT, manifesting at relatively shorter periods (above 2 s), corresponds to a brief 4-s spell of warm updrafts (that coincide with outward interactions) before being followed by another 6-s spell of warm updrafts (that coincide with ejections), as seen in Fig. 10a. Around $t\approx$ 1527:42 LT, relatively persistent cool downdrafts that manifest as high $R^2_{wT}$ at longer periods suddenly concede to short-duration warm updrafts (Fig. 10b). The relatively sudden switching causes high $R^2_{wT}$ to manifest at shorter periods (1 s and above). For 1527:42 $\le t\le$ 1528:54 LT, persistent warm updrafts in Fig. 10c contribute to high $R^2_{wT}$ for periods above 9 s, while the high $R^2_{wT}$ at shorter periods (above 2 s) is associated with the relatively shorter-duration cool downdrafts and warm updrafts (coinciding with outward interactions) that accompany the temperature microfronts. Overall, for $t\le$ 1528:54 LT, high $R^2_{wT}$ regions at periods ranging from 9 to 17 s are a manifestation of persistent cool downdrafts and persistent warm updrafts (coinciding with ejections) in Fig. 10a–c. The region of high $R^2_{wT}$ for 1528:54 $\le t\le$ 1530:42 LT is associated with the strong warming temperature microfronts passing through the mid-canopy height and canopy top in Fig. 10d, e, resulting from some of the strongest fire-induced temperature fluctuations near the canopy top (Fig. 9a). A relatively lower-frequency component of heat-flux events induced by the flame is also seen at periods ranging from 35 s to minutes. Finally, high $R^2_{wT}$ is seen within 1531:18 $\le t\le$ 1532:30 LT corresponding to cool downdrafts and warm updrafts (coinciding with outward interactions) as shown in Fig. 10f. From Fig. 11a, it appears that the tilted flame induces organized heat-flux eddies near the canopy top ranging from scales of seconds up to minute scales. We expect the organized heat-flux eddies to be associated mainly with ramps (warm-updraft events) and cliffs (cool-downdraft events) observed in the temperature signal. In that regard, it is advisable to view the scales of warm-updraft events near the canopy top in association with the range of ramp durations quantified in Sect. 4.2.1.

In contrast, regions with high $R_{wT}^2$ (within the COI) are seen sparsely at the Control Tower during this time (Fig. 11(b)) for the range of scales mentioned above. Some high-$R^2_{wT}$ regions are seen to exist at the longer periods (above 17 s and at minute scales) and the corresponding heat-flux events can be characterized as lower-frequency events. This aligns well with the observation made from Fig. 9(c) that the cooling branches of the weak temperature fronts (associated with cool downdrafts) seen in no-fire conditions persist for longer durations.

Regions of high $R_{uw}^2$ are scarce at the West Tower (Fig. 11c). This suggests that while $w_{20}$ and $T_{20}$ appear to cohere well at the West Tower in this time window, $u_{20}$ and $w_{20}$ do not seem to be well-correlated. This reflects upon the lack of variability (and organization) in momentum-flux events during this time (Fig. 10a–f) when temperature perturbations drive most of the coherent motions near the canopy top. In contrast to the West Tower, the high $R_{uw}^2$ regions for 1527:42 $\le t\le$ 1528:54 LT at the Control Tower (Fig. 11d), are associated with the persistent sweeps (of high-momentum wind) occurring near the canopy top under no-fire conditions (Fig. 9c).

It is worth looking at the cross-wavelet coherence between the heat fluxes and momentum fluxes at the West and Control Towers as shown in Fig. 11e, f, respectively. Since smoothing is accomplished in the time–frequency domain by design, we utilize $w'T'$ and $-u'w'$ rather than $\overline{w'T'}$ and $-\overline{u'w'}$ as inputs to compute the cross-wavelet coherence. Furthermore, for coherent momentum-flux events (sweeps and ejections), $u'$ and $w'$ are out of phase (by definition), implying that $-u'$ and $w'$ are in phase. In that light, we utilize $-u'w'$ instead of $u'w'$, so that regions, where coherent heat-flux events correlate well with coherent momentum-flux events, manifest as “in-phase” motions (with rightward-pointing phase arrows). It can be seen that at the West Tower, heat and momentum fluxes are relatively uncorrelated (Fig. 11e), as evidenced by the low coherence at the periods at which the heat-flux events are active. The behaviour of the heat fluxes near the canopy top is typical of free convection during which momentum-flux events are considerably suppressed. This is aligned with the increased strength and proportion of warm buoyancy-driven updrafts near the canopy top at the West Tower, as discussed earlier. Contrarily, high coherence is seen at the Control Tower for 1527:42$\le t\le$1530:06 LT, which corresponds to the duration in which cool air is imported (“swept”) into the canopy via sweeps ($-\overline{u'w'}>0$) as seen in Fig. 9(c). These sweeps are further associated with cool downdrafts ($\overline{w'T'}>0$) observed during the same time in Fig. 9(c). Moreover, $w'T'$ and $-u'w'$ are seen to be in phase during this time (rightward-pointing arrows) suggesting that the coherent heat-flux events (cool downdrafts) and coherent momentum-flux events (sweeps) during this time are well-correlated and the flow is not dominated by either of the two events.

Another approach to explore the divergence of heat and momentum fluxes from each other in the presence of a fire lies in the context of potential deviations from the Reynolds analogy (Kays et al. 1980; Li 2019). This analysis, included in the Appendix (Supplementary Information) allows us to infer the existence of strong deviations from the Reynolds analogy near the canopy top in this time window. In contrast, deviations from the Reynolds analogy near the canopy top, at the Control Tower, are found to be considerably weaker.

**Fig. 12:**
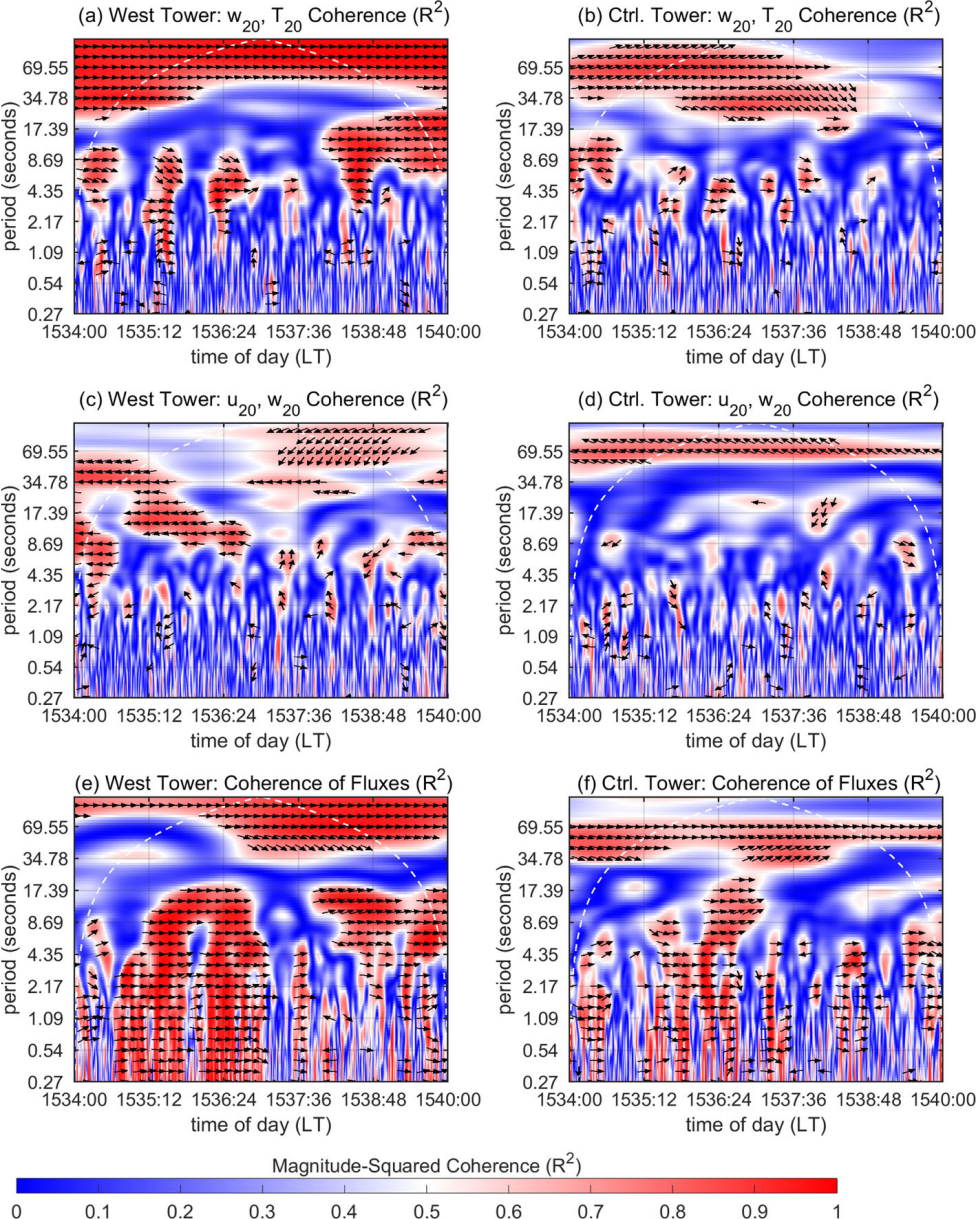
Magnitude-squared coherence ($R^2$) near the canopy top, i.e. at $h=20$ m, for the 6-min time window 1534–1540 LT. **a** $R^2_{wT}$, **c** $R^2_{uw}$, and **e** $R^2$ between $w'T'$ and $-u'w'$ at the West Tower. **b** $R^2_{wT}$, **d** $R^2_{uw}$, and **f** $R^2$ between $w'T'$ and $-u'w'$ at the Control Tower. Phase arrows are shown for $R^2\ge 0.6$ in all cases: rightward-pointing arrows indicate in-phase motions; leftward-pointing arrows indicate motions out of phase by $\pi$ radians. The white dashed line represents the cone of influence (COI) in all cases

Interesting inferences regarding the behaviour of the turbulent fluxes can be drawn from the coherence plots in the latter time window, i.e. 1534–1540 LT. At the West Tower, regions of high $R^2_{wT}$ are seen intermittently for periods ranging from 1 to 17 s (Fig. 12a), mainly representing warm updrafts (e.g., in Fig. 10g for 1534:00 $<t<$ 1534:36 LT) and cool downdrafts (e.g., in Fig. 10h–j for 1535:12 $\le t\le$ 1535:48 LT and around $t=$ 1536:24 LT) near the canopy top. Notably, the high $R_{wT}^2$ within 1538:12$<t<$1538:48 LT registers the strong warm updrafts associated with residual combustion seen in Fig. 10k. In comparison, at the Control Tower (Fig. 12b), high $R^2_{wT}$ is seen mainly at the longer periods (above 17 s and up to minutes) representing the persistent and “well-behaved” warm updrafts and the cool downdrafts accompanying the ensuing longer-duration weak cooling temperature fronts seen in Fig. 9d.

**Fig. 13:**
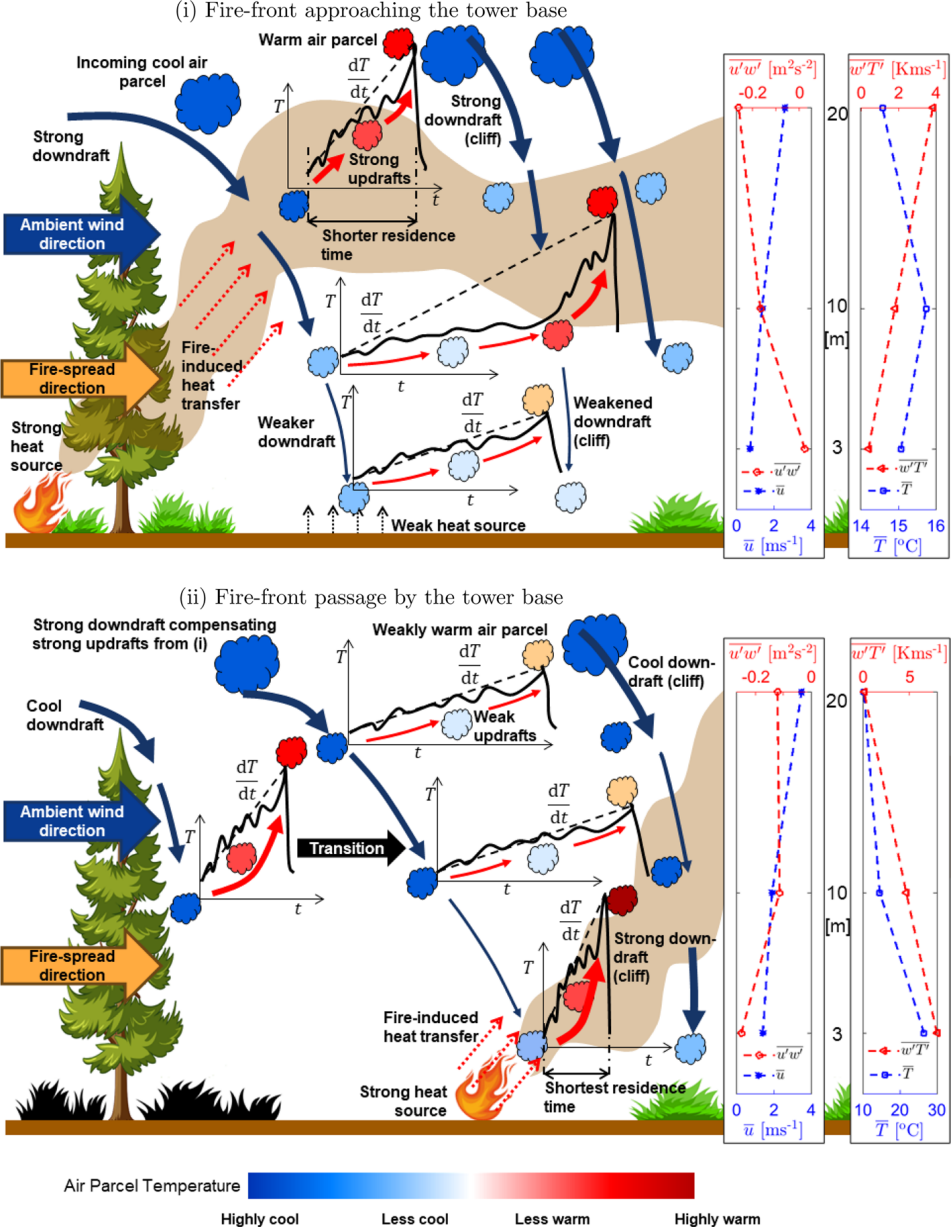
A schematic representation summarizing modulations induced by a heading sub-canopy surface fire in the ramp slope, ramp duration, and cliff slopes (i) in the earlier time window (as the fire-front approaches) and (ii) in the latter time window (during FFP) from a Lagrangian perspective (inspired by Figure 6.9 of the work by Katul et al. 2013) along with representative profiles of the mean streamwise velocity (${\overline{u}}$), turbulent momentum flux ($\overline{u'w'}$), mean temperature (${\overline{T}}$), and turbulent heat flux ($\overline{w'T'}$). Solid arrows represent local wind motion: thicker arrows represent stronger draughts while thinner arrows represent weaker draughts; dotted arrows represent heat transfer; colorbar, arrow length, and arrow thickness are not to scale; images of the flame and vegetation are taken from *www.vecteezy.com*

Contrary to the earlier time window, regions of high $R_{uw}^2$ are seen at the West Tower (Fig. 12c) at periods ranging from 2 to 35 s and above (within the COI). The (leftward-pointing) phase arrows in these regions indicate that the *u* and *w* signals are out of phase suggesting that these regions represent either ejections (e.g., in Fig. 10g for $t<$1534:36 LT) or sweeps. Most notable is the region of high $R_{uw}^2$ spanning periods ranging from 8 to 25 s for 1534:36$<t\le$1536:24 LT, which corresponds to the persistent sweeps highlighted in Fig. 10h–j. Sweeps are then registered by regions of slightly lower coherence ($0.5\le R_{uw}^2\le 0.6$) at periods above 17 s for 1536:24$\le t\le$1538:12 LT. Unlike the West Tower, regions of high coherence ($R^2_{uw}\ge 0.6$) are only seen at periods of minutes and longer at the Control Tower across the entire time window, within the COI (Fig. 12d). These are associated with the background (atmospheric) canopy turbulence.

At both towers, the coherent heat-flux events and coherent momentum-flux events appear to correlate well with each other in this time window (Fig. 12e, f). In particular, the region of high coherence for 1534:36 $\le t\le$ 1537:00 LT at the West Tower (Fig. 12e), corresponds to the regime where cool downdrafts and sweeps are well-correlated. This suggests a relatively more congruous interaction between fire-induced flow and the background atmospheric eddies compared to the earlier time window. It appears that the influx of cool ambient air into the canopy via persistent sweeps, which are momentum-flux events, is partially a response to the outflux of warm air from the canopy due to strong warm updrafts (heat-flux events) in the earlier time window. Such an influx coincides with a duration when the influence of the flame near the canopy top is relatively diminished and strong high-temperature fluctuations are only experienced near the canopy top intermittently, hindering the organization of heat-flux eddies.

We note that it is in this later time window that an enhanced organization of heat-flux-bearing eddies is observed extensively at $h=3$ m as evidenced by the cross-wavelet coherence computed at that height (Appendix). During this time, momentum-flux-bearing eddies are relatively less extensively organized, both in time and across time scales (under 1 min) near the surface. Additionally, strong deviations from the Reynolds analogy are observed near the surface. Among all heights, these deviations are found to be the strongest near the surface, due to its maximum proximity to the flame. Deviations from the Reynolds analogy at the mid-canopy height are comparatively moderated by the competing interplay between fire-induced heat fluxes and ambient momentum fluxes in both time windows. More details on the analysis leading to these inferences can be found in the Appendix (Supplementary Information). At this point, the readers are also advised that it is worth comparing our results regarding the turbulent flux events obtained from the wavelet-based analysis above against those presented in Fig. 1 for context and consistency.

We now take the opportunity to summarize our results pertaining to the effects that the presence of a heading surface fire has at different heights within the canopy at the different stages of fire-front propagation with the help of Fig. 13. As shown in Fig. 13, the tilting of the flame-front in the direction of the wind imposes a temporal lag in the modulation of ramp–cliff structures at different heights, as it does on the enhanced organization of heat-flux eddies, under the influence of the fire. We have, therefore, organized our findings under two stages: (i) as the fire-front approaches the tower and (ii) as the fire-front passes by the tower. The schematic representation encapsulates the steeper escalations to high temperatures along ramps over shorter durations, under the influence of the buoyant plume (and its stronger warm updrafts) at the higher heights, especially near the canopy top, as the fire approaches; this effect is present to a higher degree at the lower heights, especially near the surface, during the passage of the fire-front by the tower base, due to the closer proximity with the flame. At the mid-canopy height, ramp–cliff structures are first affected by the competing effects of ambient turbulence and the buoyant plume (Fig. 13i) and then predominantly by the stronger warm updrafts induced due to the closer proximity of the flame (Fig. 13ii). Moreover, the drop in temperature along a cliff at all heights is steeper and associated with stronger cool downdrafts compared to no-fire conditions (of which, the coherent structures near the surface in Fig 13i are an approximate representation). These modulations in the ramp–cliff structures observed in the temperature signal are accompanied by a change in the degree of organization of flux-bearing eddies: heat-flux eddies are well-organized at relatively shorter time scales while momentum-flux eddies are not. Contrarily, the degree of organization of heat-flux and momentum-flux eddies is relatively similar at these scales in the absence of a fire. Note that while we expect some quantitative variation in the amplitudes and durations of fire-modulated ramp–cliff structures for a higher-intensity heading fire, our results will remain qualitatively consistent with the narrative captured by Fig. 13.

## Conclusions and Future Work

Turbulent motions arising from the interaction of a propagating fire with its surrounding environment are organized at various scales. Unlike popular techniques that rely on averaging schemes, wavelet-based analyses can provide information regarding the range of temporal scales associated with the characteristic coherent structures as the fire-front evolves, overcoming a critical obstacle to the study of fire-induced turbulent motions. In this study, we have utilized wavelets to analyze the turbulence characteristics associated with a relatively low-intensity heading surface fire beneath the forest canopy. Measured temperature signals have been analyzed using the Mexican Hat wavelet, whereas turbulent fluxes have been analyzed using a complex wavelet, i.e. the Morlet Wavelet.

Patterns in the wavelet-based energy density plotted on a time–frequency plane allow us to identify specific events and structures inherent to the temperature signal at a given height. Patterns associated with the strongest temperature excursions near the surface allow us to track the FFP time at the tower base more clearly within the stipulated longer fire-front-passage duration, thereby informing our subsequent analysis. Furthermore, in the highest frequency band (0.1–5 Hz, which is the Nyquist frequency), some fire-induced variability is observed to be active, while the effect of atmospheric (canopy) turbulence is relatively much weaker. At these scales, intermittent burst-like behaviour characterized by irregular and rapid increments in the temperature signal are observed near the canopy top. More advanced studies along the lines of the analysis by Chowdhuri and Banerjee (2023) or Allouche et al. (2022) would be needed to quantify a metric for “burstiness” in the temperature signal.

The interaction of the fire with the canopy-induced sweep–ejection motions manifest in the ramp–cliff-type structures in the temperature signal, which are extracted using the wavelet-based approach as the fire-front approaches the measuring tower. This allows us to investigate the nature of fire–vegetation–atmosphere interaction compared to no-fire conditions and across the depth of the canopy sub-layer. We answer three overarching questions as described below.

(i) How does the presence of a fire impact the duration and amplitude of ramp–cliff patterns typically observed in the measured temperature signal? Moreover, are these impacts uniform across all heights within the canopy?

In response to Question (i), the presence of the fire generally shortens the ramp duration and enhances the amplitude of the ramp–cliff structures in the temperature signal. However, these impacts are not uniform across all heights within the canopy. They are strongest near the surface, owing to the maximum proximity to the flame, and weaken with height. Near the canopy top, the increase in ramp amplitudes results from the strong fire-induced buoyant updrafts. At the mid-canopy height, ramp durations increase in the presence of the fire due to the intermittent nature of the fire-plume–canopy interaction, while ramp amplitudes increase consistently because of fire-induced temperature excursions. At all heights, cliff slopes are steeper in the presence of the fire due to the sweeping motions associated with the strong downward entrainment of cooler ambient air into the canopy.

(ii) Does the presence of a fire enhance the degree of organization of heat- and momentum-flux-bearing eddies relative to no-fire conditions? If so, at what heights within the canopy and at which time scales?

In response to Question (ii), the presence of the fire impacts the degree of organization of turbulent eddies, associated with heat- and momentum-flux events, over a range of temporal scales (periods) at all heights during FFP. Fire-induced heat-flux events (eddies) can be coherent (organized) over shorter periods compared to no-fire conditions. Under the influence of the fire, momentum-flux events (eddies) are coherent (organized) at relatively longer periods compared to heat-flux events. These observations are consistent across all heights in the canopy.

(iii) What is the relative importance of heat-flux (thermally driven) events versus momentum-flux (mechanically driven) events at different stages of the fire-front evolution? Is the Reynolds analogy violated by the presence of a fire? If so, is it more violated near the canopy top or in the canopy subspace?

In response to Question (iii), the relative importance of heat-flux-bearing eddies versus momentum-flux-bearing eddies differs between two stages of the fire-front proximity to the tower base. The first stage comprises a brief time window prior to the arrival of the fire-front at the tower base, while the second stage comprises a later time window during which the strongest fire-induced temperature excursions are recorded at the tower base. In the earlier stage, the tilting of the flame-front in the direction of the wind causes the turbulent fluxes near the canopy top to be chiefly thermally driven while momentum-flux-bearing eddies are less energetic. This is associated with enhanced organization of heat-flux-bearing eddies across a range of time scales, representing warm updrafts (at relatively longer periods) and cool downdrafts (at shorter periods).

In contrast, during the later stage, heat-flux-bearing eddies near the canopy top are organized relatively intermittently and organized momentum-flux eddies start to become noticeable. These organized eddies are mainly associated with sweeps of high-momentum air from aloft, which coincide with cool downdrafts, at shorter periods, and in part with ejections of low-momentum air from the canopy subspace, which coincide with warm updrafts, at longer periods. Note that the dynamics of the increased organization of heat-flux-bearing eddies near the canopy top is similarly observed at the mid-canopy height and near the surface, in chronological order.

To investigate the behavioural similarity between momentum-flux and heat-flux transport, we have explored adherence to the Reynolds analogy by examining deviations in the turbulent Prandtl number from unity and theoretical predictions based on the Monin–Obukhov Similarity Theory (MOST). The Reynolds analogy is distorted by the presence of the fire. The Reynolds analogy is violated the most near the surface, particularly during the later stage of the fire-front passage, due to the maximum proximity to the flame. Deviations from the Reynolds analogy at the mid-canopy height are comparatively moderated by the competing interplay between fire-induced heat fluxes and ambient momentum fluxes. Finally, deviations from the Reynolds analogy near the canopy top are found to be the least prominent compared to the lower heights within the canopy subspace.

The analysis above provides valuable insights into the characteristic temporal scales at which fire-induced turbulent motions inform smoke dispersion and firebrand transport. The mean durations, associated with coherent structures, estimated at different heights can be used to construct length scales in the manner described by Raupach et al. (1996). Length scales thus obtained are necessary for estimating physical quantities used in popular one- and two-equation turbulence models for flow in forested environments (Katul et al. 2004). Differences in the observed behaviour of turbulent fluxes near the canopy top at the different stages of fire-front evolution discussed above also inform temporal variations in scalar transport and fire-spread behaviour. Insights obtained from such an analysis can be useful in testing and developing more sophisticated physics-based (e.g. LES) fire-spread, firebrand-transport, and smoke-dispersion models within forested environments. Improved predictive models can further assist in more informed and efficient fire-management operations.

In addition, our study illustrates the immense potential that wavelets carry in exploring fire-induced turbulence measurements. Future work may involve leveraging the distinct time–frequency localization properties of different wavelets to analyze different frequency bands of a measured quantity, i.e. to make a scale-based choice of the wavelet. For instance, the Morlet wavelet with its better frequency-localization properties can be used to analyze the higher frequency bands, while the Mexican Hat wavelet with its relatively better time-localization properties can be used to analyze the lower frequency bands. Furthermore, it is worth investigating the effect of the wavelet used on event interpretation by applying other wavelets, such as the Gaussian Wave or the first derivative of a Gaussian (Addison 2017), to the current data. Finally, wavelet-based techniques can be applied to other types of fire-spread scenarios such as grassland fires and backing surface fires beneath the canopy. A comparison between reconstructed signals corresponding to different scales for these scenarios could potentially reveal significant differences in the patterns of coherent structures on the basis of the surface-fire environment.

## Figures and Tables

**Fig. 14:**
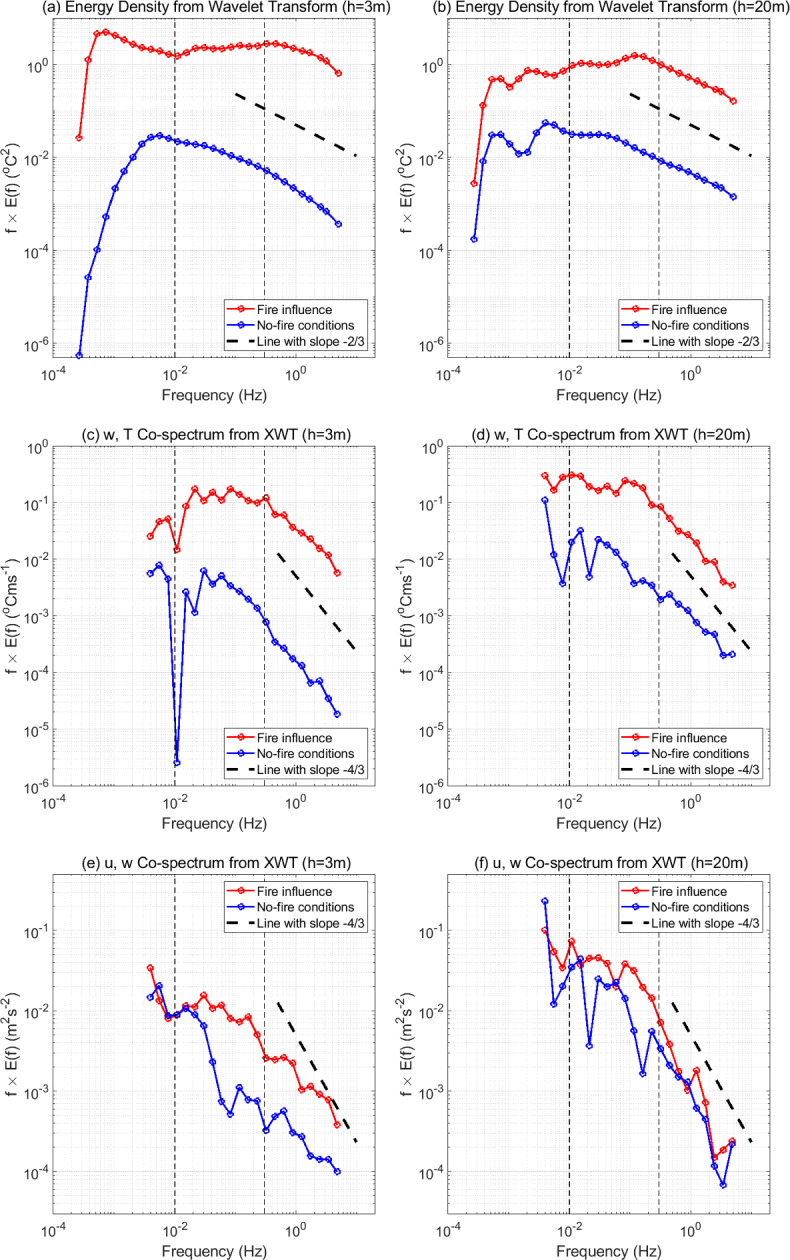
Wavelet-based pre-multiplied energy spectra and co-spectra for the 30-min period of 1520–1550 LT (5 min. pre-FFP, 20 min. during FFP, and 5 min. post-FF) at the West Tower (red) and Control Tower (blue). Pre-multiplied energy spectra (of *T*) at $h=$ **a** 3 m and **b** 20 m; *w*-*T* co-spectra at $h=$ **c** 3 m and **d** 20 m; *u*-*w* co-spectra at $h=$ **e** 3 m and **f** 20 m. Slanted thick black dashed lines have slope $-2/3$ in **a**–**b** and slope $-4/3$ in **c**–**f**; vertical black dashed lines delineate the mid-to-high frequency band within which ramp–cliff structures are tracked

**Fig. 15:**
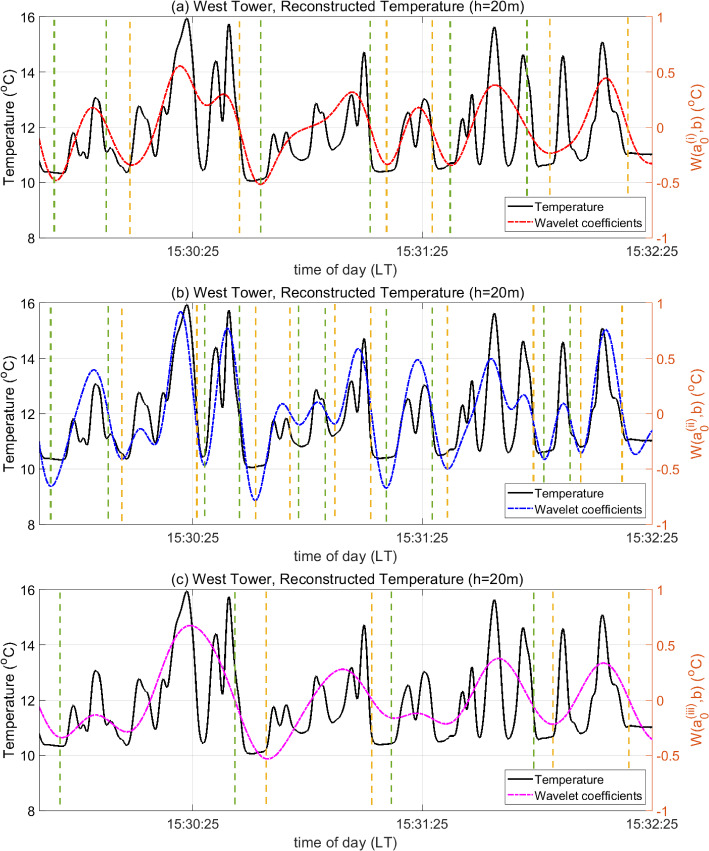
Reconstructed temperature signal at $h = 20$ m in the mid-to-high frequency band (scales ranging from 3 to 90 s) at the West Tower (where fire influence is experienced) along with the wavelet coefficients ($W_T(a_0,b)$) for (a) Case (i), (b) Case (ii), and (c) Case (iii). Dashed and dash-dotted vertical lines enclose examples of ramp-like structures: two consecutive vertical lines with the same colour and style enclose one such observed structure

**Fig. 16:**
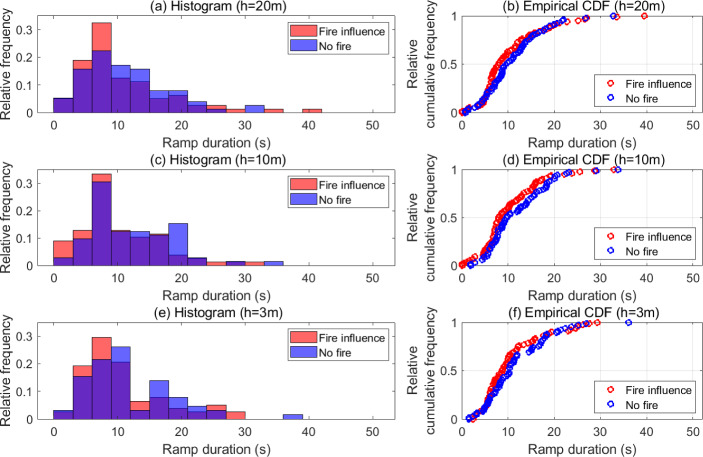
Histograms and empirical cumulative distribution functions of ramp durations associated with both, fire influence and no-fire conditions at all three measurement heights for Case (ii) ($a_0^{\text {(ii)}}\equiv f_0\approx 10/166$ Hz)

**Fig. 17:**
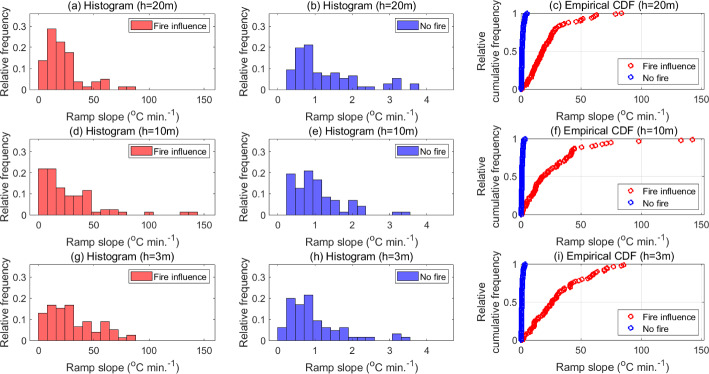
Histograms of ramp slopes associated with both, fire influence and no-fire conditions, at all three measurement heights, along with the corresponding empirical cumulative distribution functions for Case (ii) ($a_0^{{(ii)}}\equiv f_0\approx 10/166$ Hz)

**Fig. 18:**
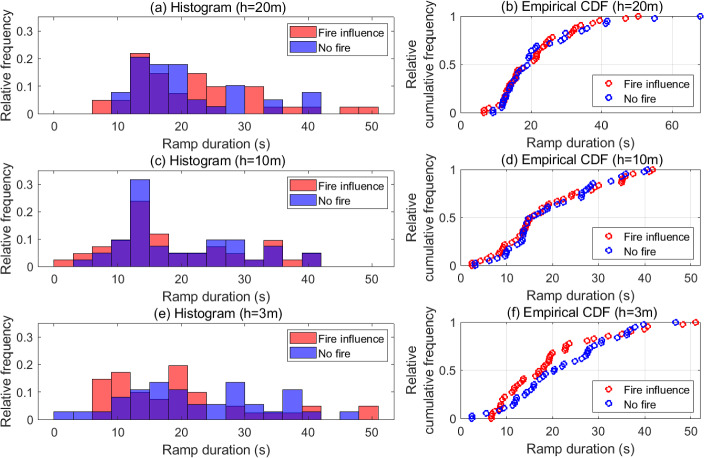
Histograms and empirical cumulative distribution functions of ramp durations associated with both, fire influence and no-fire conditions at all three measurement heights for Case (iii) ($a_0^{{(iii)}}\equiv f_0\approx 10/327$ Hz)

**Fig. 19:**
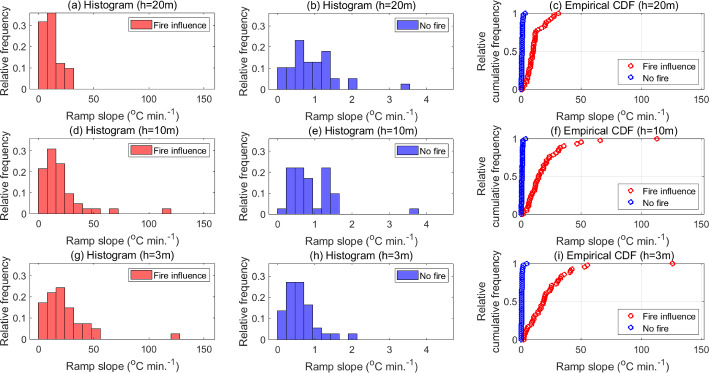
Histograms of ramp slopes associated with both, fire influence and no-fire conditions, at all three measurement heights, along with the corresponding empirical cumulative distribution functions for Case (iii) ($a_0^{(iii)}\equiv f_0\approx 10/327$ Hz)

**Fig. 20:**
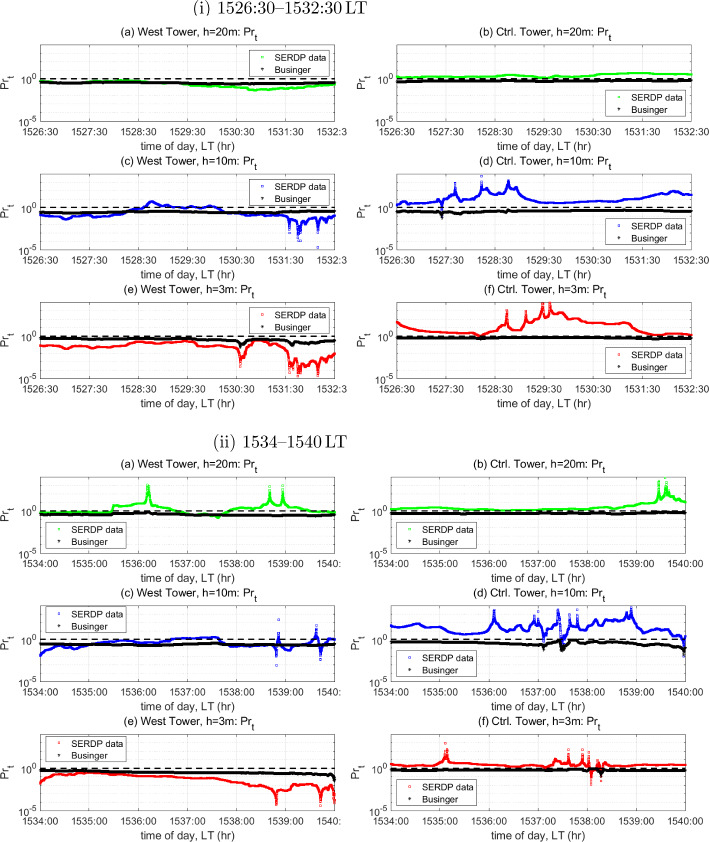
Time series of $Pr_\text {t}$ obtained from Eq. (9) and from the stability parameter ($\zeta$) using the Businger relation at the three measurement heights, at both the West Tower (under fire influence) and the Control Tower (no-fire conditions) within (i) 1526:30–1532:30 LT and (ii) 1534–1540 LT

**Fig. 21:**
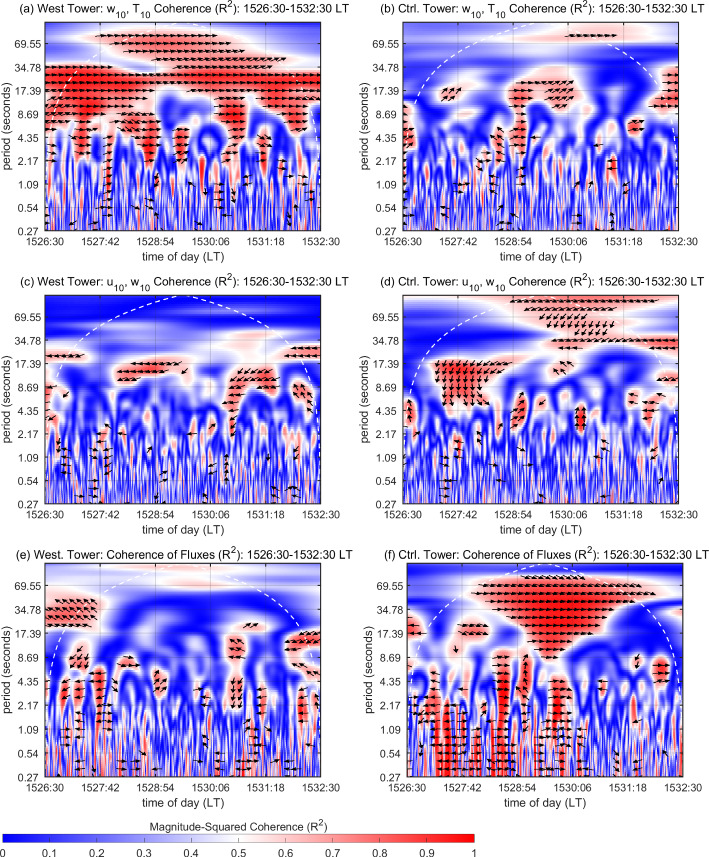
Magnitude-squared coherence ($R^2$) at the mid-canopy height, i.e. at $h=10$ m, within the 6-min time window 1526:30–1532:30 LT. **a** $R^2_{wT}$, **c** $R^2_{uw}$, and **e** $R^2$ between $w'T'$ and $-u'w'$ at the West Tower. **b** $R^2_{wT}$, **d** $R^2_{uw}$, and **f** $R^2$ between $w'T'$ and $-u'w'$ at the Control Tower. Phase arrows are shown for $R^2\ge 0.6$ in all cases: rightward-pointing arrows indicate in-phase motions; leftward-pointing arrows indicate motions out of phase by $\pi$ radians. White dashed lines represent the cone of influence (COI) in all panels

**Fig. 22:**
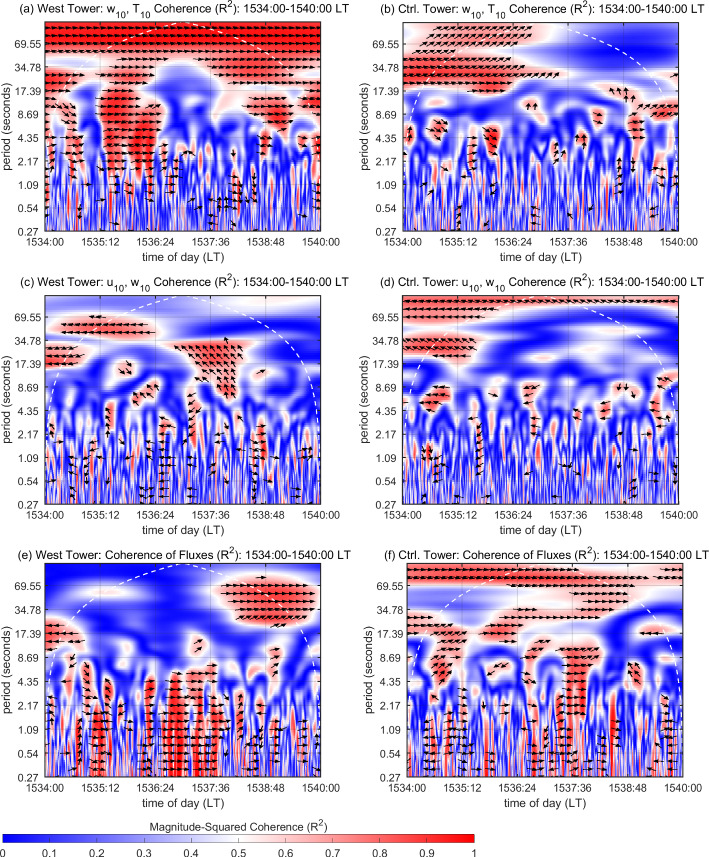
Magnitude-squared coherence ($R^2$) at the mid-canopy height, i.e. at $h=10$ m, within the 6-min time window 1534–1540 LT. **a** $R^2_{wT}$, **c** $R^2_{uw}$, and **e** $R^2$ between $w'T'$ and $-u'w'$ at the West Tower. **b** $R^2_{wT}$, **d** $R^2_{uw}$, and **f** $R^2$ between $w'T'$ and $-u'w'$ at the Control Tower. Phase arrows are shown for $R^2\ge 0.6$ in all cases: rightward-pointing arrows indicate in-phase motions; leftward-pointing arrows indicate motions out of phase by $\pi$ radians. White dashed lines represent the cone of influence (COI) in all panels

**Fig. 23:**
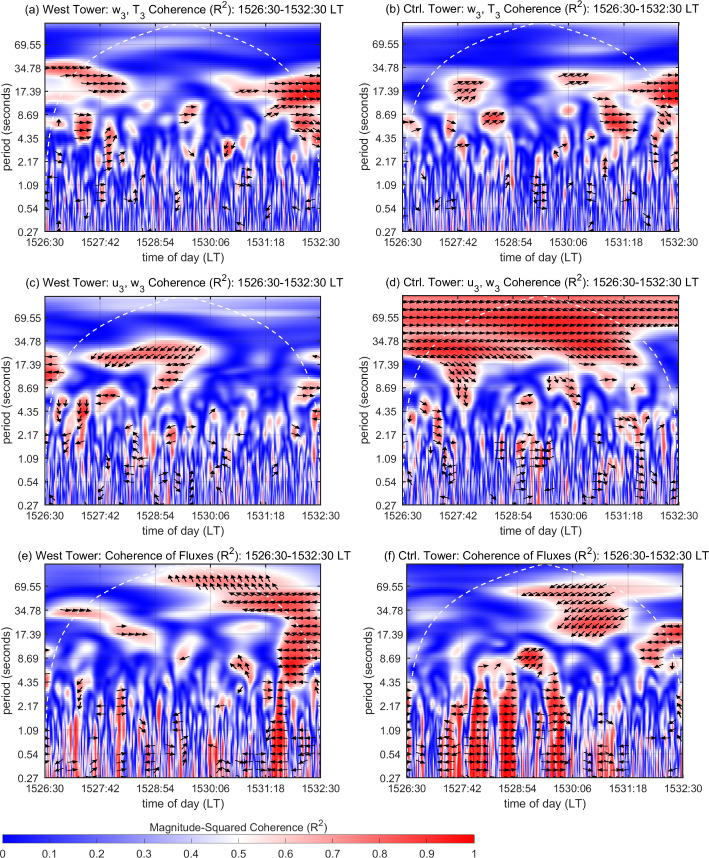
Magnitude-squared coherence ($R^2$) near the surface, i.e. at $h=3$ m, within the 6-min time window 1526:30–1532:30 LT. **a** $R^2_{wT}$, **c** $R^2_{uw}$, and **e** $R^2$ between $w'T'$ and $-u'w'$ at the West Tower. **b** $R^2_{wT}$, **d** $R^2_{uw}$, and **f** $R^2$ between $w'T'$ and $-u'w'$ at the Control Tower. Phase arrows are shown for $R^2\ge 0.6$ in all cases: rightward-pointing arrows indicate in-phase motions; leftward-pointing arrows indicate motions out of phase by $\pi$ radians. White dashed lines represent the cone of influence (COI) in all panels

**Fig. 24:**
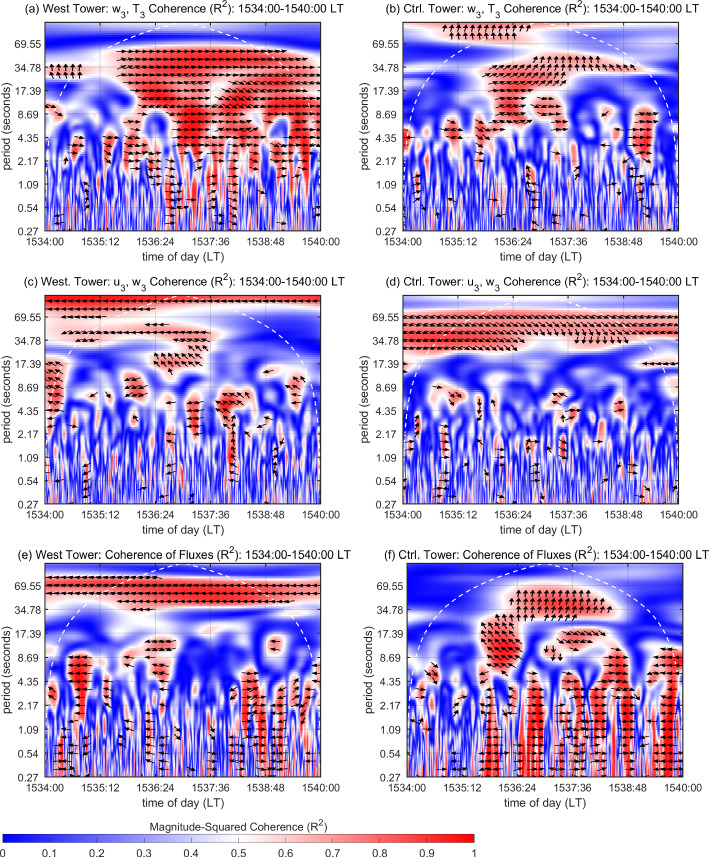
Magnitude-squared coherence ($R^2$) near the surface, i.e. at $h=3$ m, within the 6-min time window 1534–1540 LT. **a** $R^2_{wT}$, **c** $R^2_{uw}$, and **e** $R^2$ between $w'T'$ and $-u'w'$ at the West Tower. **b** $R^2_{wT}$, **d** $R^2_{uw}$, and **f** $R^2$ between $w'T'$ and $-u'w'$ at the Control Tower. Phase arrows are shown for $R^2\ge 0.6$ in all cases: rightward-pointing arrows indicate in-phase motions; leftward-pointing arrows indicate motions out of phase by $\pi$ radians. White dashed lines represent the cone of influence (COI) in all panels

## Wavelet Spectra and Co-spectra

In this section, we briefly comment on the appropriateness of the range of frequencies selected to obtain the reconstructed temperature signal within which the coherent ramp–cliff structures are tracked. This is done by analyzing the wavelet-based temperature spectra and the co-spectrum of *w* and *T*, on one hand, and that of *u* and *w*, on the other. Furthermore, we comment on the choice of the scale associated with the wavelet coefficients used to track ramp–cliff patterns ($a_0$).

The wavelet spectral energy density (*E*) as a function of scale (*a*) can be written using the following formula (Addison 2017):

4 $$\begin{aligned} E(a) = \frac{1}{C_g}\int _{-\infty }^{\infty }|W(a,b)|^2\text {d}b. \end{aligned}$$

It follows that the spectral energy density can also be represented as a function of frequency (*f*), i.e. as *E*(*f*); the spectral energy density pre-multiplied by the frequency, i.e. *fE*(*f*), represents the pre-multiplied energy spectrum.

Figure 14a, b depict the (pre-multiplied) energy spectra of the measured temperature obtained from the wavelet coefficients computed for the duration 1520–1550 LT (comprising 5 min pre-FFP, 20 min of the stipulated FFP time, and 5 min post-FFP at the West Tower) at both, the West and Control Towers. The range of frequencies, used to compute the reconstructed temperature signal to track ramp–cliff structures, is delineated using dashed black vertical lines. It is observed that the spectral energy associated with the West Tower is higher compared to that for the Control Tower, at both heights. This is attributed to the higher variability in the measured temperature during FFP as compared to the measured ambient temperature at both heights. Furthermore, the influence of the fire being stronger near the surface due to higher proximity, the spectral energy is relatively higher at $h=3$ m compared to at $h=20$ m, at the West Tower.

We first focus on the peak frequencies for both towers at $h=3$ m (Fig. 14a). At the West Tower, it is seen that the peak frequency ($f_\text {p}$) is approximately $6\times 10^{-4}$ Hz, while $f_\text {p}\approx 5\times 10^{-3}$ Hz at the Control Tower. The shift of $f_\text {p}$ from a higher value in no-fire conditions to a lower value during FFP suggests that the most energetic turbulent eddies induced by the presence of the fire are associated with relatively lower frequencies (or longer time scales) compared to those affiliated with the background canopy turbulence near the surface. Moreover, a few additional peaks are observed within the range $10^{-2}$–0.3 Hz, at the West Tower.

We now focus on the peak frequencies of the temperature spectra at $h=20$ m (Fig. 14b). At both towers, it is seen that $f_\text {p}\approx 5\times 10^{-4}$ Hz. Furthermore, a secondary peak is seen at $f\approx 2\times 10^{-3}$ Hz at the West Tower, while a secondary peak is seen at $f\approx 4\times 10^{-3}$ Hz, at the Control Tower. Again, a few additional peaks are observed within the range $10^{-2}$–0.3 Hz, at the West Tower.

At the West Tower, the peak frequencies reported above can be used to compute peak time scales ($t_\text {p}$). Peak time scales are within the range 1600–2000 s for both $T_{3}$ and $T_{20}$, while the secondary peak for $T_{20}$ corresponds to 500 s. Each of these peak time scales is much longer compared to the durations of approximately 1–2 min that are typically associated with ramp–cliff structures in no-fire conditions (Katul et al. 2013). Since ramp–cliff structures are expected to have even shorter durations in the presence of a fire, we reconstruct the temperature signals using a range of periods (3–90 s) that encompass the additional peaks in the energy spectra of the temperature at the two heights, at the West Tower. These periods being much shorter than the peak time scales obtained from the energy spectra, it is difficult to use the wavelet coefficients at periods corresponding to $t_\text {p}$ for the purpose of quantifying the durations and slopes of the ramp structures within the reconstructed temperature signal as described in Sect. 3.2.2. Therefore, in our analysis, we have chosen $a_0$ so that the wavelet coefficients associated with this scale are able to track the pattern of the coherent ramp–cliff structures from the local minima and zero-crossings as accurately as possible, as verified from a visual inspection ($a_0\equiv 23.3$ s or $f_0\approx 10/233$ Hz). Since this introduces some uncertainty in the estimates of the ramp durations and slopes, we conduct an analysis that explores the sensitivity of these quantities to different values of $a_0$ in the next section.

We now focus on the wavelet co-spectra. The wavelet co-spectrum between two time series *p*(*t*) and *q*(*t*) is computed from the cross-wavelet transform using the following formula:

5 $$\begin{aligned} E(a) = \frac{1}{C_g}\int _{-\infty }^{\infty }\Re \left[ W_{pq}(a,b))\right] \text {d}b. \end{aligned}$$

Figure 14c–f depict the wavelet co-spectra computed for the duration 1520–1550 LT at both, the West and Control Towers. Figure 14c, d represent the wavelet co-spectra of *w* and *T*, pre-multiplied with *f*, at $h=$3 m and 20 m, respectively. It is observed that the range of frequencies within which we track fire-modulated ramp–cliff structures in the (reconstructed) temperature signal encompasses the peak frequencies associated with the “energetic” heat fluxes under the influence of the fire (at the West Tower), prior to the onset of the inertial subrange. We then analyze momentum fluxes in the same range of frequencies (delineated in Fig. 14e, f) for consistency. Therefore, it is suitable to track the coherent ramp–cliff structures and explore their connection to the turbulent fluxes in the presence of a fire within this range of frequencies ($10^{-2}$–0.33 Hz).

## Sensitivity Analysis of Ramp Durations and Slopes

The statistics of ramp durations and slopes for three different scale parameters (or frequencies) are analyzed here. Of these, we refer to the case for $a_0$ corresponding to $f_0\approx 10/233$ Hz as Case (i), which serves as our reference case. The statistics corresponding to Case (i) are documented in Sect. 4.2 and are not repeated here. Cases (ii) and (iii) are summarized in Table 3.

**Table 3:** A summary of the time scales and frequencies used in the sensitivity analysis

Case No.	Scale parameter	Time scale (period) (s)	Frequency ($f_0$) (Hz)
(i)	$a_0^{\text {(i)}}$	23.3	10/233
(ii)	$a_0^{\text {(ii)}}$	16.6	10/166
(iii)	$a_0^{\text {(iii)}}$	32.7	10/327

Here, $a_0^\text {(ii)}< a_0^\text {(i)}< a_0^\text {(iii)}$. Figure 15 depicts the reconstructed temperature signal at the West Tower for $h=20$ m along with the time series of the wavelet coefficients ($W_T(a_0,b)$), for the three cases discussed in Table 3, within a short time window. Dashed vertical lines indicate the locations of local minima and zero-crossings with negative slopes in $W_T(a_0,b)$ for each case. Two consecutive dashed vertical lines of the same colour delineate individual ramp-like structures as detected by the algorithm. It can be seen that in Case (ii), the algorithm is more likely to detect short-scale temperature increments and declines, which constitute coherent ramp–cliff structures, as individual ramp–cliff structures in themselves. On the contrary, in Case (iii), the relatively shorter-scale temperature increments and declines that characterize the shorter-scale coherent events during FFP are overlooked by the algorithm in favor of relatively longer-scale changes in temperature. The effects of the differences in ramp detection on the statistics of the ramp durations and slopes in each case are presented below.

### Case (ii)

Figure 16 depicts the histograms and empirical cumulative distribution functions (eCDFs) for Case (ii), while the associated summary statistics are shown in Table 4. The modal durations (as seen from the histograms in Fig. 16) for fire-modulated ramps are shorter than 10 s at all heights. At all three heights, the percentage of both fire-modulated and no-fire ramps with durations not exceeding 10 s is higher compared to that in Case (i), as observed from the eCDFs. The modes of the histograms at $h=10$ m, 20 m are shifted to the left compared to the histograms for Case (i), in both the presence of the fire and no-fire conditions. At $h=3$ m, 20 m, the mean durations for fire-modulated ramps are approximately 3 s shorter compared to those in Case (i), while the mean durations for no-fire ramps are approximately 6 s shorter than those in Case (i), as seen from Table 4. This difference is explained as follows. The caveat of using shorter-scale ($a_0^{\text {(ii)}}$) wavelet coefficients in Case (ii) is that shorter-scale temperature increments and declines, which “ride” (or constitute) the relatively longer-scale coherent ramp–cliff structures, are counted by the algorithm as individual ramp–cliff structures. This results in an underestimation of ramp durations compared to those obtained using relatively longer-scale wavelet coefficients in Case (i) (i.e. $W_T(a_0^\text {(i)},b)$). This further explains why the median ramp durations as seen from the eCDFs (Fig. 16) are lower for both the fire-modulated and no-fire ramps at all heights in comparison with the median ramp durations for Case (i).

It would seem that the statistics for the ramp durations in Case (ii) provide some evidence to support our hypothesis that fire-modulated (West Tower) ramp durations are shorter compared to no-fire (Control Tower) ramps. At all three heights, the mean durations for fire-modulated ramps are shorter compared to those for no-fire conditions (Table 4). While the histogram modes for fire-modulated and no-fire ramps appear to coincide near the canopy top and at the mid-canopy height (Fig. 16), the most probable durations for fire-modulated ramps are shorter than those for no-fire ramps near the flame, i.e. at $h=3$ m. The eCDFs show that there is a higher percentage of ramps with durations shorter than 10 s under fire influence in comparison with no-fire conditions at all three heights. The median fire-modulated ramp duration (where the relative cumulative frequency is 0.5) is shorter compared to the median no-fire ramp duration at all heights. While it appears that the durations of fire-modulated ramps are shorter than those of no-fire ramps, the difference is not as pronounced as in Case (i).

**Table 4:** Height-wise summary statistics of fire-modulated ramps and ramps in no-fire conditions for Case (ii) ($a_0^{\text {(ii)}}\equiv f_0\approx 10/166$ Hz). Modes are obtained directly from the data (not from the histograms)

Height (*h*) (m)	Fire-modulated ramps					No-fire ramps				
	No. sampled	Mean duration (s)	Modal duration (s)	95th percentile duration (s)	Mean slope ($^{\circ }\text {C}\,\textrm{min}^{-1}$)	No. sampled	Mean duration (s)	Modal duration (s)	95th percentile duration (s)	Mean slope ($^{\circ }\,\text {C} \textrm{min}^{-1}$)
20	80	10.5	6.3	26.0	22.5	76	11.3	4.3	21.9	1.4
10	78	10.3	6.8	23.2	28.6	72	12.0	6.0	22.5	1.1
3	78	10.6	5.5	25.0	37.4	65	11.8	10.4	24.1	1.0

Next, Fig. 17 shows the histograms and eCDFs of ramp slopes computed at all three heights. As seen from the eCDFs, the percentage of fire-modulated ramps exceeding 50 $^{\circ }$C min$^{-1}$ is higher for Case (ii) as compared to Case (i). This happens because the algorithm computes the slope of the shorter-scale temperature increments mentioned above instead of the slope of a more coherent ramp structure. Moreover, shorter-scale temperature increments are associated with shorter durations. Ramp slopes, therefore, have a tendency to be relatively overestimated. This is also reflected in the mean fire-modulated ramp slopes at all heights (Table 4), which are overestimated compared to mean fire-modulated ramp slopes in Case (i). Ramp slopes in no-fire conditions are also slightly overestimated in comparison with those in Case (i). Nevertheless, in Case (ii), the mean fire-modulated ramp slope is an order of magnitude higher than the mean slope of a ramp in no-fire conditions (Table 4), which is an inference similar to that obtained in Case (i).

#### Case (iii)

Figure 18 depicts the histograms and eCDFs of ramp durations at all three heights for Case (iii), while Table 5 provides some of the associated summary statistics. It can be seen that the modes of these histograms are shifted to the right compared to histograms for Case (i). The most probable durations for fire-modulated ramps, as seen from the histograms, are longer than 10 s (and longer compared to that for Case (i)) at all heights. As seen from Table 5, the mean durations for fire-modulated ramps are overestimated (longer) compared to those in Case (i). This difference is more palpable at $h=3$ m (approximately 6 s) and at $h=20$ m (approximately 7.5 s). Similarly, the mean durations for no-fire ramps are also overestimated compared to those in Case (i) at $h=3$ m and 20 m by approximately 5 s. This difference is explained as follows. The issue in using longer-scale wavelet coefficients ($W_T(a_0^{{(iii)}},b)$), as in this case, is the opposite of that discussed in Case (ii) above: relatively shorter-scale ramp–cliff structures are overlooked by the algorithm. Consecutive coherent ramp–cliff structures are together counted as one structure resulting in an overestimation of the average duration of a ramp in both fire and no-fire conditions. This further explains why the median ramp durations as seen from the eCDFs (Fig. 18) for both fire-modulated and no-fire ramps are longer than those observed for Case (i).

**Table 5:** Height-wise summary statistics of fire-modulated ramps and ramps in no-fire conditions for Case (iii) ($a_0^{\text {(iii)}}\equiv f_0\approx 10/327$ Hz). Modes are obtained directly from the data (not from the histograms)

Height (*h*) (m)	Fire-modulated ramps					No-fire ramps				
	No. sampled	Mean duration (s)	Modal duration (s)	95th percentile duration (s)	Mean slope ($^{\circ }\,\text {C}$ $\textrm{min}^{-1}$)	No. sampled	Mean duration (s)	Modal duration (s)	95th percentile duration (s)	Mean slope ($^{\circ }\text {C}$ $\textrm{min}^{-1}$)
20	41	21.5	20.8	42.8	11.7	39	22.9	19.4	48.9	0.9
10	42	19.0	12.8	38.8	20.7	41	19.6	14.3	37.6	0.9
3	41	19.7	9.1	44.1	23.2	37	22.3	17.2	39.1	0.7

We now compare the statistics of the fire-modulated and no-fire ramp durations for Case (iii). The percentage of fire-modulated ramps with durations shorter than 20 s is higher than that of ramps in no-fire conditions at $h=3$ m, i.e. near the flame (as seen from the eCDFs in Fig. 18). Moreover, the median fire-modulated ramp duration is noticeably shorter compared to the median ramp duration in no-fire conditions, at this height. Similar to Case (i), the mean fire-modulated ramp duration is higher than the mean ramp duration in no-fire conditions at $h=3$ m and 20 m (Table 5). The difference, however, is less than 1.5 s and not as pronounced as in Case (i). Furthermore, the eCDFs for fire and no-fire conditions at higher heights, i.e. at $h=20$ m and $h=10$ m (Fig. 18b, d), overlap with each other to a considerable extent, such that the median ramp durations appear to be similar at each of those heights. The explanation for this is similar to that provided in the previous paragraph. Ramp–cliff structures are closely related to the updraft-downdraft events that occur near the canopy top. Longer-scale wavelet coefficients ($W(a_0^{(iii)}, b)$) are unable to capture the effects of the more frequent, characteristic shorter-scale updraft–downdraft events that occur near the canopy top as a consequence of fire-front-passage (FFP). This results in the statistics of the fire-modulated ramp durations being very similar to those of the ambient, no-fire ramp durations.

Next, Fig. 19 shows the histograms and eCDFs of ramp slopes computed at all three heights for Case (iii). It appears from the eCDF at $h=3$ m (Fig. 19i) that the percentage of fire-modulated ramp slopes above 50 $^{\circ }$C$\,\text {min}^{-1}$ is lower compared to that in Case (i). As seen from Table 5, both mean fire-modulated and no-fire ramp slopes are underestimated compared to those computed in Case (i). At $h=10$ m, mean ramp slopes in both categories are underestimated by approximately 10%. Mean fire-modulated ramp slopes are underestimated by approximately 26% and mean no-fire ramp slopes by 18% at $h=20$ m. Again, this is because the algorithm utilizes the temperature differences over longer time durations to compute the slope, while omitting the relatively shorter-scale increments that characterize fire-modulated ramps. However, the mean fire-modulated ramp slope is an order of magnitude higher than that in no-fire conditions (Table 5), which is an inference similar to that obtained in Case (i).

It appears that the choice of the scale parameter (or frequency) associated with the wavelet coefficients used to detect ramp-like structures influences the average ramp durations computed at all three heights. This appears to hold true for both fire-modulated as well as ambient (no-fire) ramps. In all the cases considered here, the mean duration is consistently found to be shorter for fire-modulated ramps as compared to ambient (no-fire) ramps both near the canopy top and near the flame. The difference, however, is most pronounced when wavelet coefficients corresponding to Case (i) are used. For instance, the mean fire-modulated ramp durations at $h=3$ m are shorter than ambient (no-fire) ramp durations by approximately 10% in Case (ii) and 12% in Case (iii). However, this difference corresponds to approximately 22% in Case (i). This is because longer-scale wavelet coefficients (Case (iii)) are unable to capture the shorter-scale updraft downdraft events that characterize the ramp–cliff structures modulated by the fire presence; on the other hand, shorter-scale wavelet coefficients (Case (ii)) detect (often erroneously) less coherent shorter-scale events as coherent ramp-like structures in both ambient conditions as well as in the presence of a fire. A more rigorous method may be needed for selecting a suitable scale parameter for analyzing ramp-like structures. Across all three cases, however, it must be noted that mean fire-modulated ramp slopes are higher by an order of magnitude than mean ambient (no-fire) ramp slopes. In all three cases, the ratio of the mean fire-modulated ramp slope to the mean ambient ramp slope is consistently above 30 at $h=3$ m and ranges from 13 to 17 at $h=20$ m. Thus, while we need to be careful in selecting the appropriate scale for the wavelet coefficients being used to detect ramp-like structures in the temperature signal, the method shows considerable promise in estimating the important mean statistics that encapsulate the changes occurring in ramp–cliff structures (coherent motions beneath the canopy) due to the presence of the fire at the forest surface and the buoyant plume at the higher heights.

## Estimating Sensible Heat Fluxes from Ramps

In this section, we demonstrate the applicability of ramp–cliff patterns observed in measured temperature signals to compute sensible heat fluxes in the presence of a fire, by invoking the surface renewal method (e.g., Paw et al. 1995; Katul et al. 1996; Fischer et al. 2023). Sensible heat fluxes can be computed using the following formula (Katul et al. 2013):

6 $$\begin{aligned} H = \rho c_\text {p}\frac{\text {d} T}{\text {d} t}\frac{V}{A}. \end{aligned}$$

Here $\text {d} T/\text {d} t$ is the time rate of temperature change from a Lagrangian perspective, $c_\text {p}$ is the specific heat capacity of air (approximately $1004\,\hbox {J}\,{\textrm{kg}}^{-1}\hbox {K}^{-1}$), *V* is the air-parcel volume (associated with the ramp) and *A* is the ground area. Furthermore, d*T*/d*t* is given by (Katul et al. 2013):

7 $$\begin{aligned} \frac{\text {d}T}{\text {d}t} = \frac{\partial T}{\partial t} + u_i\frac{\partial T}{\partial x_i} = \frac{\partial T}{\partial t} + \frac{\partial u_i T}{\partial x_i}\approx \frac{\partial T}{\partial t}. \end{aligned}$$

Here the latter approximation is typically justified in no-fire conditions by arguing that the interaction between $u_i$ and *T* is manifested at higher frequencies compared to those at which coherent structures (such as ramps) are observed in the temperature signal (*T*) (Katul et al. 2013; Fischer et al. 2023; Paw et al. 1995). In the presence of a fire, however, it is difficult to ignore the advection term ($\partial (u_iT)/\partial x_i$) since gradients in $u_iT$ are expected to be stronger. For instance, $\partial (u_iT)/\partial x$ is expected to be substantial across the flame due to the fire-induced acceleration of the local wind (Desai et al. 2023). It is reasonable to assume that the contribution of both components of the Lagrangian derivative (d*T*/d*t*), i.e. the local time derivative ($\partial T/\partial t$) and the advection term, to the sensible heat fluxes, is significant. However, the absence of data collection in the horizontal direction in this experiment hinders the computation of some of these partial derivatives ($\partial /\partial x$ and $\partial /\partial y$ terms). Moreover, data collection may be needed at additional heights to quantify vertical derivatives ($\partial /\partial z$ terms) more accurately as well, at each height. Therefore, we focus on estimating the contribution due to local changes in temperature, at this stage.

The contribution to the sensible heat flux due to local changes in the temperature ($H_\text {ramp}$) can be estimated from the ramp slope ($\partial T/\partial t$) in the presence of a fire using the following formula:

8 $$\begin{aligned} H_\text {ramp} = \rho c_\text {p}\frac{\partial T}{\partial t}\frac{V}{A}. \end{aligned}$$

Typically, it is assumed that *V*/*A* is proportional to the canopy height $h_\text {c}$, i.e. $V/A= \alpha h_\text {c}$, where $\alpha$ is a scalar constant (Fischer et al. 2023). The strongest turbulent eddies resulting in the steepest ramp–cliff patterns impacted by the fire (flame or plume) can be assumed to influence almost the entire canopy along the vertical axis, based on the warmest temperature isotherms shown in Fig. 9 of the main text. Therefore, it is reasonable to assume $\alpha =1$, i.e. $V/A \approx h_\text {c}$, at all heights within the canopy. Setting $\partial T/\partial t$ to the value of the mean ramp slope at each height, we obtain values for $H_\text {ramp}$ as shown in Table 6. In the presence of a fire, it can be observed that $H_\text {ramp}$ is highest near the surface and decreases with height due to decreasing proximity with the heat source (the flame) with height. Contrarily, in the absence of a fire, $H_\text {ramp}$ increases with height since the influx of ambient heat from the atmospheric surface layer aloft (and through incoming solar radiation) is highest near the canopy top.

**Table 6:** Height-wise summary of sensible heat fluxes derived from fire-modulated ramps and ramps in no-fire conditions

Height (*h*) (m)	Fire-modulated ramps			No-fire ramps		
	Mean slope ($^\circ \text {C}$ $\textrm{min}^{-1}$)	$H_\text {ramp}$ (W $\textrm{m}^{-2}$)	$H_\text {turb}$ (W $\textrm{m}^{-2}$)	Mean slope ($^\circ \text {C}$ $\mathrm{min.}^{-1}$)	$H_\text {ramp}$ (W $\textrm{m}^{-2}$)	$H_\text {turb}$ (W $\textrm{m}^{-2}$)
20	15.8	5298	4869	1.1	369	289
10	18.8	6304	7135	1.0	335	185
3	27.2	9121	8476	0.8	268	51

For comparison, we have tabulated the turbulent sensible heat fluxes ($H_\text {turb}$) derived from the kinematic heat fluxes ($\overline{w'T'}$) using the formula: $H_\text {turb} = \rho c_\text {p} \overline{w'T'}$ (Table 6). The kinematic heat fluxes are obtained from 2-min moving averages (represented by the overbar operator) within the corresponding time window during which fire-modulated ramps were tracked at each height. These are similar to the heat fluxes presented in Fig. 1. It can be observed that the values of $H_\text {turb}$ and $H_\text {ramp}$ are comparable under the influence of the fire, while these values diverge at lower heights within the canopy (by approximately 81% at $h=10$ m and more than 400% at $h=3$ m) in the absence of a fire. One limitation of this comparison descends from the fact that the values of the kinematic heat flux ($\overline{w'T'}$) are sensitive to the averaging time window used to compute them. Moreover, as discussed above, we have not quantified the contribution to the sensible heat fluxes due to the advection terms. Nevertheless, our estimates capture the inversion in the vertical profile of the sensible heat flux (from no-fire conditions) under the influence of the surface fire within the canopy with an error of less than 12% at all heights. Thus, the analysis demonstrates that the application of ramp–cliff patterns in estimating sensible heat fluxes through the surface renewal method, in the presence of a fire within a forest canopy, is promising.

## Deviation from the Reynolds Analogy

Adherence to the Reynolds analogy (Kays et al. 1980) is typically explored from values of the turbulent Prandtl number ($Pr_\text {t}$), where $Pr_\text {t}=\nu _\text {t}/\alpha _\text {t}$. Here, $\nu _\text {t}$ represents the turbulent momentum diffusivity and $\alpha _\text {t}$ represents the turbulent diffusivity of heat. When the Reynolds analogy holds, $Pr_\text {t}\rightarrow 1$, which suggests a similarity between turbulent momentum fluxes and turbulent heat fluxes. However, differences in the behaviour of heat and momentum fluxes manifest as a deviation of $Pr_\text {t}$ from 1 and represent a distortion of the Reynolds analogy. Alternatively, $Pr_\text {t}$ can also be expressed in terms of the stability correction functions, $\phi _\text {h}$ and $\phi _\text {m}$, conceived by Monin and Obukhov (1954), as shown below (Li 2019):

9 $$\begin{aligned} \phi _\text {m}= &  \frac{\kappa z}{u_*}\frac{\partial {\overline{u}}}{\partial z}, \phi _\text {h} = \frac{\kappa z}{T_*}\frac{\partial {\overline{T}}}{\partial z}, \text {where}~u_*^2 = -\overline{u'w'}~\text {and}~T_*= \frac{-\overline{w'T'}}{u_*} \nonumber \\ Pr_\text {t}= &  \frac{\phi _\text {h}}{\phi _\text {m}} = \frac{u_*}{T_*}\frac{\partial {\overline{T}}}{\partial z}\left( \frac{\partial {\overline{u}}}{\partial z}\right) ^{-1} = \frac{\overline{u'w'}}{\overline{w'T'}}\frac{\partial {\overline{T}}}{\partial z}\left( \frac{\partial {\overline{u}}}{\partial z}\right) ^{-1}. \end{aligned}$$

Here the overbar operator represents a 2-min moving mean, and $\kappa$ is the von Kármán constant. Note that the right-hand side (RHS) of Eq. (9) is equivalent to the ratio of the turbulent diffusivities ($\nu _\text {t}/\alpha _\text {t}$) as discussed above.

Previous studies (e.g., McColl et al. 2017) have alluded to deviation from the Reynolds analogy in unstable atmospheric conditions: $Pr_\text {t}$ decreases ($Pr_\text {t}<1$) as atmospheric conditions become more strongly unstable (Li 2019), as would be expected in the presence of a fire. In that light, we explore the time series of $Pr_\text {t}$ at each measurement height, at both the West and Control Towers, for deviations from unity within the two sub-durations (i) 1526:30–1532:30 LT (Fig. 20(i)) and (ii) 1534–1540 LT (Fig. 20(ii)). We have utilized the mean quantities and turbulent fluxes shown in Fig. 1 to compute $Pr_\text {t}$ from Eq. (9). Note that we have taken the absolute value of the expression on the RHS of Eq. (9) to avoid negative values of $Pr_\text {t}$ that arise from the non-monotonic nature of the mean temperature profile within the canopy subspace (Figs. 1 and 13).

In the earlier time window (as the fire-front approaches), deviations from the Reynolds analogy are observed near the canopy top ($h=20$ m) at the West Tower (Fig. 20(i)a). This is seen particularly after 1529:30 LT ($Pr_\text {t}<1$ and $Pr_\text {t}\rightarrow 0.05$) as heat fluxes become stronger near the canopy top due to the influence of the tilted flame and buoyant plume. After 1530 LT, strong deviations from the Reynolds analogy are also observed at the mid-canopy height (Fig. 20(i)c) for reasons akin to those near the canopy top, while the strong deviations near the surface (Fig. 20(i)e) occur not only due to weak momentum fluxes (Figs. 1(i)b and 13(i)) but also due to changes induced in the mean temperature profile within the canopy subspace ($\partial {\overline{T}}/\partial z \rightarrow 0$, as seen from Fig. 1(i)c) by the approaching fire-front. In contrast, deviations from the Reynolds analogy near the canopy top in no-fire conditions (Fig. 20(i)b) are relatively weaker ($1\le Pr_\text {t}\le 4.5$). Additionally, the behaviour of the time series of $Pr_\text {t}$ near the surface and at the mid-canopy height in no-fire conditions (Fig. 20(i)d, f), differs considerably from that at the West Tower, at these heights.

In the later time window (as the fire-front passes by), deviations from the Reynolds analogy are observed mid-canopy and near the surface at the West Tower (Fig. 20(ii)c, e, respectively). At $h=3$ m, deviations from the Reynolds analogy start to become stronger ($Pr_\text {t}<<1$) after 1536 LT as heat fluxes at this height become stronger (Fig. 1(ii)c) due to maximum proximity to the flame. Deviations from the Reynolds analogy are observed earlier on at $h=10$ m ($Pr_\text {t}<1$ before 1535:30 LT), which get weaker in time as the deviations at $h=3$ m continue to grow stronger. It appears that deviations at the mid-canopy height are weaker compared to those observed near the surface due to the competing effects of fire-induced heat fluxes and ambient momentum fluxes at the mid-canopy height. Near the canopy top (Fig. 20(ii)a), deviations from the Reynolds analogy are observed from 1535:30–1536:30 LT; however, $Pr_\text {t}>>1$, suggesting that this deviation is attributed to the relatively stronger momentum fluxes compared to heat fluxes during this time.

Note that this time interval (1535:30–1536:30 LT) corresponds to the duration when persistent sweeping motions are observed and momentum-flux events gain prominence near the canopy top (Fig. 9). In no-fire conditions (at the Control Tower), it is observed that $1\le Pr_\text {t}\le 2.5$ for the most part (before 1538 LT) near the canopy top, suggesting that deviations from the Reynolds analogy are comparatively weaker here, in this time duration (Fig 20(ii)b). Again, the behaviour of the time series of $Pr_\text {t}$ near the surface and at the mid-canopy height in no-fire conditions (Fig. 20(ii)d, f), differs considerably from that at the West Tower, at these heights.

The Monin–Obukhov Similarity Theory (MOST) also allows us to express the correction functions in stably or unstably stratified conditions in the ASL as functions of the stability parameter (e.g., Businger et al. 1971; Dyer 1974, etc.). Therefore, we can obtain $Pr_\text {t}$ as a function of the stability parameter (thermal stratification) using the Businger relation as follows (Businger et al. 1971; Li 2019):

10 $$\begin{aligned} \begin{aligned} Pr_\text {t}&= 0.74 \frac{(1-9\zeta )^{-\frac{1}{2}}}{(1-15\zeta )^{-\frac{1}{4}}}, \zeta < 0 \\&= \frac{0.74+4.7\zeta }{1+4.7\zeta }, \zeta >0. \end{aligned} \end{aligned}$$

The stability parameter ($\zeta$) in Eq. (10) can be expressed in terms of the Obukhov length (*L*) (Kaimal and Finnigan 1994) as follows:

11 $$\begin{aligned} \zeta = \frac{z}{L},~\text {where}~L = \frac{-u_*^3}{\kappa }\frac{{\overline{T}}}{g\overline{w'T'}}. \end{aligned}$$

A comparison between predictions of $Pr_\text {t}$ from the Businger relation (Eq. 10) and values of $Pr_\text {t}$ computed directly from the correction functions (Eq. 9) can be accomplished in the $Pr_\text {t}$–$\zeta$ plane for unstable conditions ($\zeta <0$) as well as stable conditions ($\zeta >0$) within each time window (e.g., Li 2019). Our analysis (not shown here) revealed significant distortions from the predictions made by the Businger relation both in the absence and presence of a surface fire within the canopy; these distortions were notably significant during unstable conditions at all three heights at the West Tower, i.e. in the presence of the fire, in both time windows.

Here, we compare the predictions to computed values of $Pr_\text {t}$ by representing them as a time series, as shown in Fig. 20. It is observed that predictions from the Businger relation bear some resemblance to the values of $Pr_\text {t}$ from Eq. (9), at $h=20$ m, as the fire-front approaches (Fig 20(i)a). However, the relation is unable to predict the diminished values of $Pr_\text {t}$ ($Pr_\text {t}\rightarrow 0.05$) attained when departures from the Reynolds analogy become stronger near the canopy top (after 1530 LT in Fig. 20(i)a). Moreover, the Businger relation is unable to predict the large values of $Pr_\text {t}$ attained within the later time window when momentum fluxes become relatively stronger near the canopy top (1535:30–1536:30 LT in Fig. 20(ii)a). In the absence of a fire (at the Control Tower), predictions from the Businger relation and computed values of $Pr_\text {t}$ represented as a time series near the canopy top appear to be somewhat comparable in both time windows (Figs. 20(i)b, (ii)b).

Within the canopy subspace ($h=3$ m, 10 m), significant departures are observed between predictions made by the Businger relation and computed values of $Pr_\text {t}$ represented as a time series, both in the presence and absence of a fire (West and Control Towers, respectively), in both time windows (Fig. 20(i)(c)–(f), (ii)(c)–(f)). Near the surface, predictions are substantially overestimated compared to computed values of $Pr_\text {t}$ at the West Tower in both time windows (Fig. 20(i)e, (ii)e). Contrarily, in the absence of a fire (at the Control Tower), predictions generally underestimate the computed values of $Pr_\text {t}$ in both time windows, particularly within the canopy subspace.

This analysis reveals that predictions from the functional forms of the $Pr_\text {t}$ in terms of the stability parameter (the Businger relation, in this case) are not always consistent with the values of $Pr_\text {t}$ obtained from the turbulent fluxes and vertical gradients in the mean temperature and streamwise velocity (Eq. 9) in the canopy sublayer, both in the presence and absence of a fire. These inconsistencies may emanate from the underlying assumptions involved in the MOST, which generally limits its application within plant canopies.

In summary, the Reynolds analogy is distorted by the presence of the fire. This occurs at different time instances near the surface, mid-canopy, and near the canopy top, attributed mainly to the increased strength of turbulent heat fluxes, as the fire-front advances towards and passes by the measuring tower. The Reynolds analogy is violated the most near the surface due to maximum proximity to the flame, particularly during the later stage of the fire-front passage. Deviations from the Reynolds analogy at the mid-canopy height are comparatively moderated by the competing interplay between fire-induced heat fluxes and ambient momentum fluxes. Deviations from the Reynolds analogy near the canopy top are found to be the least intense compared to the lower heights within the canopy subspace. It must be noted that our analysis is based on the Monin–Obukhov Similarity Theory (MOST), which relies on certain assumptions so that its validity remains to be explored within plant canopies, both in the presence and absence of a surface fire. This constitutes a limitation of this analysis and presents a potential subject for future research.

## Cross-Wavelet Coherence in Canopy Subspace

Cross-wavelet coherence analysis allows us to comment on the degree of organization of turbulent eddies in the presence of a fire. We have already provided detailed insights on the association of the cross-wavelet coherence with the temperature isotherms and flow near the canopy top in Sect. 4.3.2. Here, we extend the analysis to briefly explore changes in the organization of turbulent eddies at the mid-canopy height and near the surface in both time windows, based on the cross-wavelet coherence, associated with heat and momentum fluxes ($R^2_{wT}$ and $R^2_{uw}$, respectively), depicted in Figs. 21, 22, 23 and 24.

The presence of the fire results in the organization of flux-bearing eddies at the West Tower, particularly for heat flux, within a range of frequencies or periods. Such organization is observed in different time durations at different heights. The increased organization of heat-flux eddies within a range of periods (1–35 s and frequently up to 1 min.) is seen earliest near the canopy top (consistently within 1528:54–1531:18 LT) due to the tilting of the flame in the direction of the wind (Fig. 11). Within the same range of periods, such behaviour is then chronologically observed at the mid-canopy height (intermittently within 1527:42–1531:18 LT and consistently within 1535:12–1536:24 LT as observed from Figs. 21a and 22a, respectively), followed by the near-surface height (1536:24 LT onwards as observed from Fig. 24a). The organized (coherent) heat-flux events comprise cool downdrafts or warm updrafts.

The organization of heat-flux eddies at the measurement heights, under the influence of a fire (at the West Tower), is more noticeable compared to momentum-flux eddies from the relatively more extensive regions of high $R^2_{wT}$ within one of (or both) the time windows (depending on the height). This suggests two interrelated possibilities: the presence of the flame or the buoyant plume either (I) does not enhance the organization of momentum-flux eddies at shorter periods (below 35 s) as it does for the heat-flux eddies or (II) destroys pre-existing coherence, if any, in ambient momentum-flux events at shorter periods.

Possibility (II) can be explored via an analysis of $R^2_{uw}$ in no-fire conditions. At the Control Tower, regions of high coherence ($R^2_{uw}$) are seen at relatively higher periods near the surface (35–60 s in Fig. 23d and 17–60 s in Fig. 24d). The (rightward-pointing) phase arrows in the regions of high $R^2_{uw}$ at $h=3$ m in both time windows indicate that *u* and *w* are not out of phase when the turbulent momentum-flux-bearing eddies are well-organized near the surface. This suggests that the most coherent momentum-flux events near the surface in the absence of the fire are counter-gradient motions ($\overline{u'w'}>0$, as seen in Fig. 1(i)b, (ii)b) rather than sweeps or ejections. In the presence of a fire (at the West Tower), however, coherent momentum-flux eddies associated with “in-phase” (counter-gradient) *u*–*w* motions are scarce near the surface (Figs. 23c and 24c). In fact, organized momentum-flux-bearing eddies, associated with the albeit fewer regions of high $R^2_{uw}$ in the presence of a fire, are observed to manifest as either sweeps or ejections as discerned from the (leftward-pointing) phase arrows at the West Tower. Therefore, it appears that the presence of a fire disorganizes the pre-existing coherent momentum-flux events comprising counter-gradient motions near the surface, in this range of periods, and reorganizes them as sweeps or ejections, albeit relatively less extensively than heat-flux events.

Finally, it must be noted that regions of high coherence between heat fluxes, on one hand, and momentum fluxes, on the other, are found to be more extensive in no-fire conditions (at the Control Tower) compared to the West Tower, at the mid-canopy height and near the surface. This is seen by comparing panel (e) to panel (f) in each of Figs. 21, 22, 23 and 24. The higher coherence between heat and momentum fluxes across a range of periods suggests that these fluxes are comparatively more similar in behaviour, in no-fire conditions.
